# Metabolic and macrovascular outcomes following simultaneous pancreas–kidney transplantation and kidney transplantation alone in recipients with type 1 diabetes mellitus: a systematic review and meta-analysis

**DOI:** 10.1007/s00125-026-06843-x

**Published:** 2026-08-29

**Authors:** Harsham Choksi, Rowan Klein Nulend, Tess Cooper, Eric Au, Henry Pleass, Natasha M. Rogers

**Affiliations:** 1 Centre for Transplant and Renal Research, Westmead Institute for Medical Research, Westmead, NSW Australia; 2 Faculty of Medicine and Health, The University of Sydney, Camperdown, NSW Australia; 3 Department of Surgery, Westmead Hospital, Westmead, NSW Australia; 4 Cochrane Kidney & Transplant, Centre for Kidney Research, The Children’s Hospital at Westmead, Westmead, NSW Australia; 5 Sydney School of Public Health, The University of Sydney, Camperdown, NSW Australia; 6 Melbourne School of Population and Global Health, University of Melbourne, Parkville, VIC Australia; 7 Renal and Transplantation Medicine, Westmead Hospital, Westmead, NSW Australia

**Keywords:** Cardiovascular disease, Cerebrovascular disease, Glycaemic management, Kidney transplantation, Lipid profile, Macrovascular disease, Meta-analysis, Metabolic outcomes, Peripheral vascular disease, Simultaneous pancreas–kidney transplantation, Systematic review, Type 1 diabetes

## Abstract

**Aims/hypothesis:**

Simultaneous pancreas–kidney transplantation (SPKTx) and kidney transplantation alone (KTx) are common treatment modalities for individuals with type 1 diabetes with end-stage kidney disease. This study compared metabolic and macrovascular disease outcomes between SPKTx and KTx in recipients with type 1 diabetes.

**Methods:**

We conducted a systematic search of the Embase, MEDLINE and Scopus databases (last searched 23 February 2025). We included retrospective and case–control studies, owing to a lack of randomised controlled trials in English, with adult patients. We excluded studies reporting on a return to dialysis post transplant, islet–kidney transplantation or separate pancreas–kidney transplants. Bias and study quality were assessed via the Newcastle–Ottawa scale for cohort studies. Demographic and outcome data were synthesised as both categorical and continuous data, with pooled means and summary statistics computed where applicable. Meta-analysis was performed on post-transplant outcomes in studies evaluating total cholesterol (TC), triglyceride (TG) level, HDL, LDL, blood pressure, HbA_1c_ and renal function. Macrovascular outcomes included the frequency of new or adverse events associated with coronary artery disease (CAD), cerebrovascular disease (CeVD) and peripheral vascular disease (PVD).

**Results:**

A total of 15 studies (*n*=564 SPKTx, 419 KTx) were included. Meta-analysis demonstrated lower post-transplant TC (mean difference −0.41 mmol/l [95% CI −0.60, −0.22], *p*<0.01, *I*^2^=37%), TG level (mean difference −0.76 mmol/l [95% CI −1.02, −0.49], *p*<0.01, *I*^2^=86%), LDL (mean difference −0.28 mmol/l [95% CI −0.42, −0.14], *p*<0.01, *I*^2^=15%), HbA_1c_ (mean difference −28.79 mmol/mol [95% CI −35.75, −21.82], *p*<0.01, *I*^2^=98%; mean difference −2.61% [95% CI −3.05, −2.16], *p*<0.01) and creatinine levels (mean difference −21.54 μmol/l [95% CI −39.23, −3.85], *p*=0.02, *I*^2^=90%) following SPKTx compared with KTx. Mean antihypertensive agent use per participant decreased post SPKTx (1.0 vs 2.5 pre transplant; *p*<0.01), but was not significantly different from post-KTx groups. HbA_1c_ increased following KTx (69 mmol/mol [8.5%]) compared with pre KTx (53 mmol/mol [7.0%], *p*<0.01). Progression of CAD, CeVD and PVD occurred across both groups post transplant. PVD showed more than a twofold increase in amputations and revascularisations post transplant. No difference was observed in the post-transplant frequency of CAD, CeVD or PVD between SPKTx and KTx. Evidence from this study was limited by a lack of subgroup data for analysis, a lack of randomised studies and a paucity of reported lifestyle factors influencing outcomes.

**Conclusions/interpretation:**

SPKTx demonstrates a superior metabolic profile post transplant to that in KTx in recipients with type 1 diabetes. However, this does not translate into differences in the risk of CAD or PVD post transplant, with PVD accounting for the greatest burden of macrovascular disease.

**Registration:**

This protocol has been registered a priori on PROSPERO (CRD42024622408).

**Graphical Abstract:**

**Figure:**
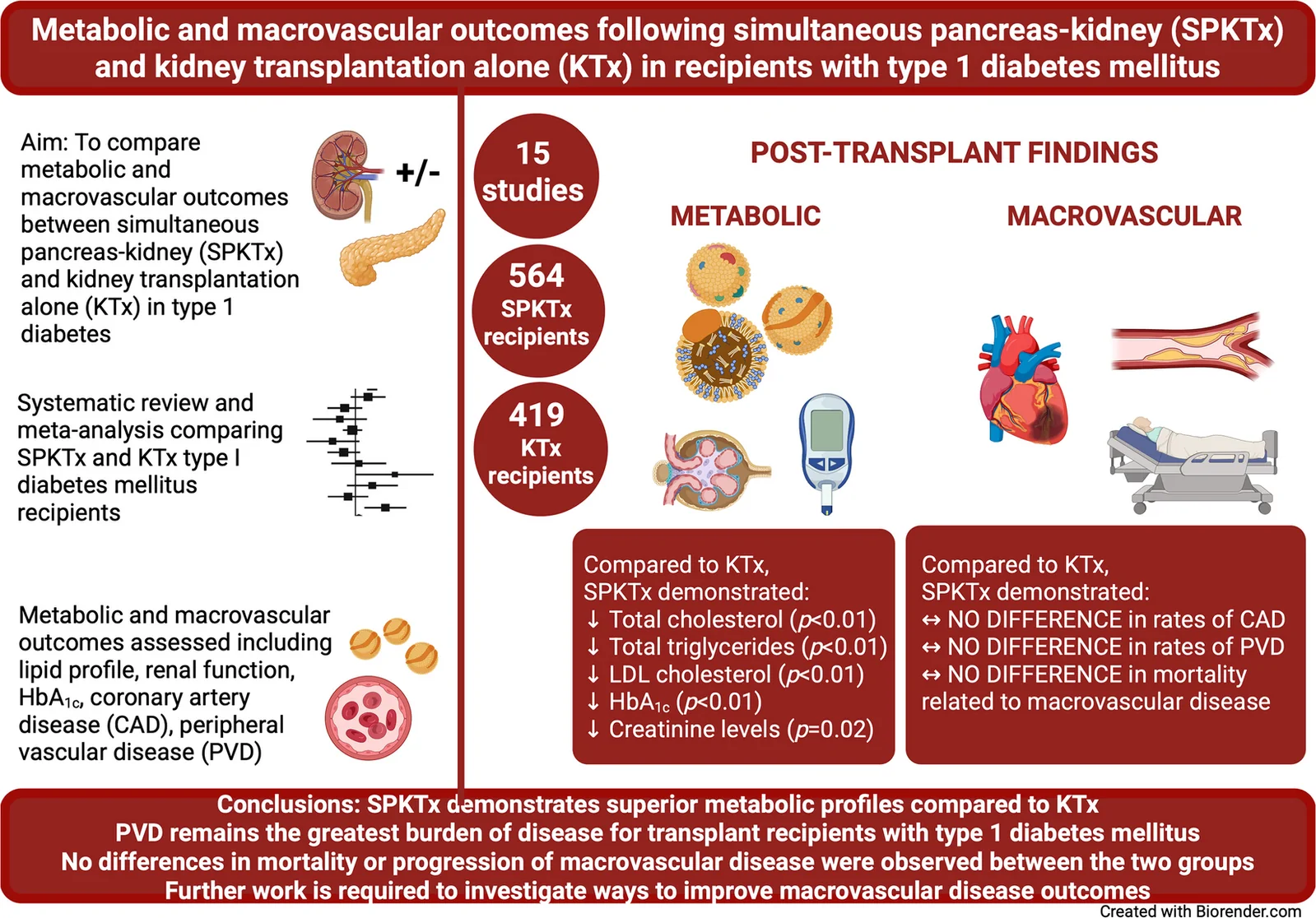

**Supplementary Information:**

The online version contains peer-reviewed but unedited supplementary material available at https://doi.org/10.1007/s00125-026-06843-x.

**Figure:**
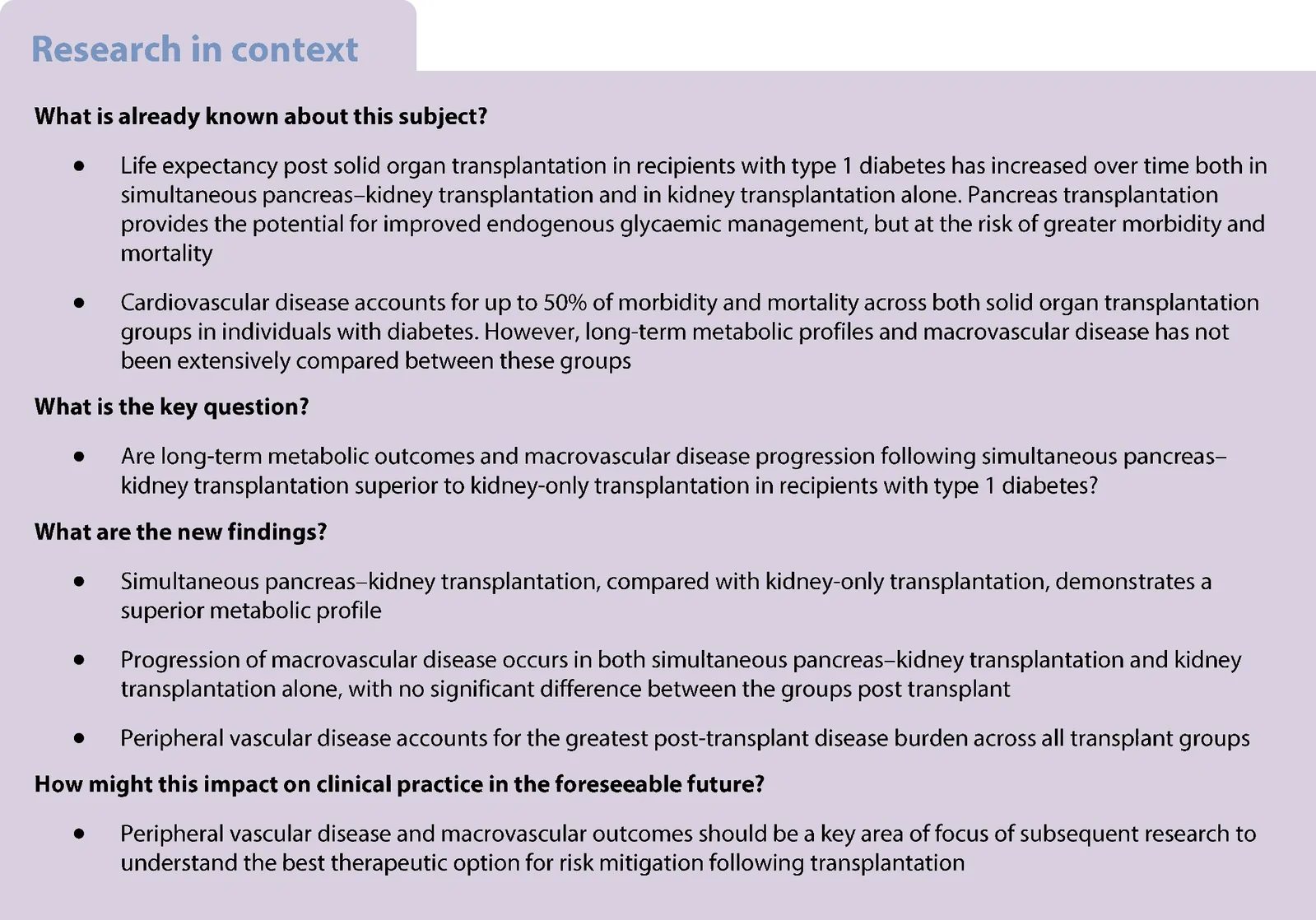

## Introduction

Type 1 diabetes mellitus is a chronic disease characterised by absolute dependence on exogenous insulin and, despite rigorous treatment, contributes to significant morbidity and mortality [1]. Complications include microvascular disease (peripheral neuropathy, nephropathy and retinopathy) and macrovascular disease (coronary artery disease [CAD], peripheral vascular disease [PVD] and cerebrovascular disease [CeVD]) [2]. Although pharmacological and technical advances have improved insulin synthesis, delivery and titration, it is not possible to match the endogenous glycaemic management achieved in healthy individuals. End-stage kidney disease (ESKD) resulting from type 1 diabetes often manifests with cardiovascular disease and hypertension, increasing the burden of comorbid disease [3]. In younger individuals with type 1 diabetes and concurrent ESKD, dialysis is an unsustainable long-term solution due to significant morbidity and mortality risk, particularly compared with organ transplantation [4].

Simultaneous pancreas–kidney transplantation (SPKTx) and kidney transplantation alone (KTx) are the most common solid organ transplantation options for individuals with type 1 diabetes with ESKD [5]. SPKTx provides superior outcomes [6] and has potentially shorter waiting times, although KTx remains an alternative option for management, with considerations surrounding peri-operative recipient fitness and procedural complications (including pancreatic allograft loss) and patient preference contributing to decision-making [7]. While both modalities demonstrate excellent 5-year graft survival outcomes, kidney grafts from live donor kidney recipients perform better than those acquired from deceased donors [8], while a long-term survival benefit is associated with a functioning pancreatic allograft in SPKTx [9].

In recent decades, patient and graft survival in both SPKTx [10] and KTx [11] recipients has improved, probably due to a combination of reduced technical failures (including superior surgical expertise and enteric drainage of pancreatic allografts) [12], a more sophisticated assessment of immunological risk and refined immunosuppressive protocols (particularly widespread use of T cell-depleting agents, tacrolimus and mycophenolic acid) [13]. However, cardiovascular morbidity—likely exacerbated by concurrent metabolic derangement seen post transplantation [14, 15]—accounts for up to 50% of deaths post transplantation, highlighting the need to focus on metabolic outcomes and cardiovascular risk management to prolong recipient life expectancy [16]. Evaluating differences between SPKTx and KTx with regard to long-term glycaemic management, lipid profile changes and renal function can inform how morbidity following transplantation may be reduced. Given the chronicity of type 1 diabetes, recipients often have evidence of pre-existing micro- and macrovascular disease that can be worsened by glycaemic derangement due to immunosuppression use. Both calcineurin inhibitor (CNI) and mammalian target of rapamycin inhibitor (mTORi) lead to beta cell dysfunction [17] and insulin resistance [18, 19]. Therefore, a robust understanding of changes in vascular disease with transplantation can provide valuable data regarding modality choice and benefits of beta cell replacement. The objectives of this study were to evaluate metabolic and cardiovascular risk factors following SPKTx or KTx in individuals with type 1 diabetes and to compare vascular disease progression between the two groups.

## Methods

### Search strategy and data extraction

The protocol used in this review was registered a priori on PROSPERO (CRD42024622408). In accordance with the Preferred Reporting Items for Systematic Reviews and Meta-Analyses (PRISMA) guidelines, we conducted a systematic literature search of the Embase, MEDLINE and Scopus databases from inception to 23 February 2025 (electronic supplementary material [ESM] Table 1) [20]. Studies reporting metabolic and macrovascular disease outcomes following either KTx or SPKTx in individuals with type 1 diabetes were selected. The full search strategy is outlined in ESM Table 2. Full text articles were reviewed independently for eligibility by HC and RKN, with inconsistencies resolved through consensus. Studies were also identified through citations from relevant articles and scoping searches of reviews discussing metabolic outcomes in transplant recipients.

### Study selection

Due to a lack of randomised controlled trials and prospective studies, we included retrospective cohort and case–control studies in our review, while excluding case series, case reports, abstracts, conference proceedings and non-peer-reviewed publications. We restricted studies to those in English and to those involving only adult recipients (>18 years). Studies with a minimum follow-up of 6 months post transplant and pre-emptive or live donor transplants were also included. We excluded studies reporting a return to dialysis post transplant, non-functioning allografts, on islet–kidney transplantation, on pancreas-after-kidney transplantation and on type 2 diabetes cohorts.

### Bias appraisal

Bias and the quality of studies were evaluated using the Newcastle–Ottawa Scale (NOS) for cohort studies [21]. Preliminary assessments were made by HC and any discrepancies were clarified with RKN and TC. Papers adjudged to be of poor quality or with a very high risk of bias (≤3 on the NOS) were excluded from the analysis. Differences in any assessments by authors were managed by discussion and mutual agreement.

### Outcomes of interest

Outcomes of interest included post-transplant indicators of metabolic health including creatinine levels, antihypertensive medication use, glycated haemoglobin (HbA_1c_) and lipid profile measures such as total cholesterol (TC), triglyceride (TG) levels, HDL and LDL. Comparative studies reporting pre- and post-transplant incidence or events associated with macrovascular disease such as PVD, CeVD or CAD were also analysed. We defined ‘progression of macrovascular complications’ on follow-up as new or worsening disease identified through radiographic or objective patient assessment, those requiring revascularisation or intervention post transplant, or any adverse events or new diagnoses including but not limited to myocardial infarction, cerebrovascular accidents or amputations. Demographic data for SPKTx and KTx populations were also collected, including age at transplant, proportion of female recipients, mean duration of follow-up, and mean duration of dialysis and type 1 diabetes prior to transplantation. Patterns of immunosuppression medication use were collected where reported. Data from eligible full text studies were extracted by HC and checked by RKN and TC prior to data analysis.

### Data synthesis and analysis

We quantitatively synthesised demographic and outcome data. Categorical data were presented as frequency and percentage. Continuous data were presented as mean ± SD unless otherwise specified. Where possible, pooled means and summary statistics were computed. We performed meta-analyses using the Review Manager 5 software [22] (accessed 1 February 2026). Effect measures were reported as odds ratios (for categorical variables) or mean difference (for continuous variables) where applicable. Pooled estimates were calculated using random-effects models. All effect measures for continuous data were computed from reported post-intervention values, not change from baseline. A separate analysis was conducted for pre- and post-transplant outcomes. Subgroup analyses by age, sex, pre-emptive transplantation, duration of dialysis or type 1 diabetes, and immunosuppression regimen could not be conducted owing to a lack of data. Heterogeneity was assessed via the *I*^2^ statistic [23] and individual studies contributing to high heterogeneity were excluded for sensitivity analyses. We excluded data that had been incompletely reported.

A ‘summary of findings’ (SoF) table was generated to outline the magnitude of the effect for each meta-analysed outcome, with the certainty of evidence ascertained using the five GRADE (Grades of Recommendation, Assessment, Development and Evaluation) considerations, including the risk of bias, the consistency of the effect, imprecision, indirectness and publication bias [24].

## Results

### Search results and assessment of bias

**Fig. 1:**
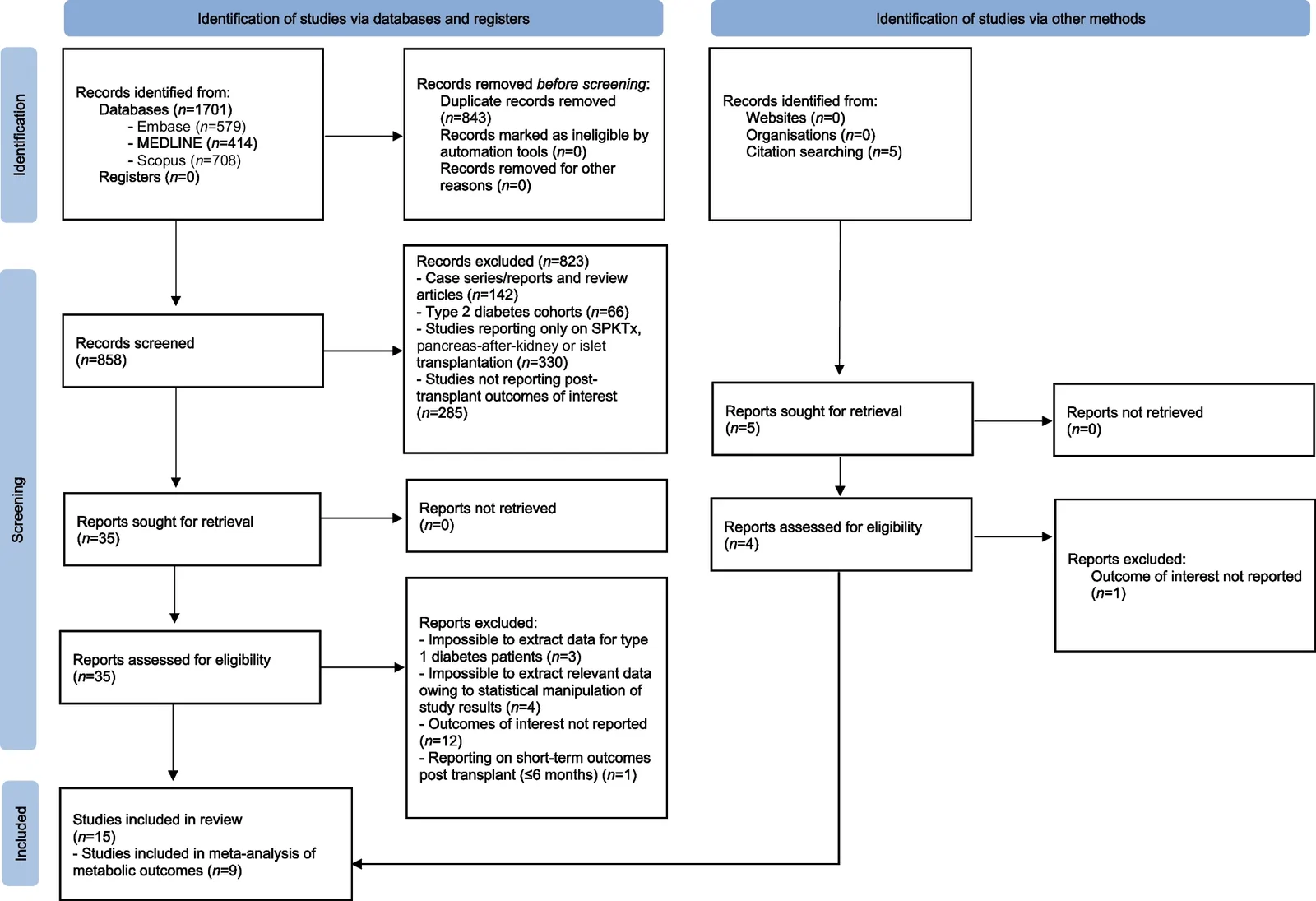
PRISMA flow diagram for the process of study selection

Our systematic literature search yielded 15 studies with 564 SPKTx and 419 KTx recipients [25–39]. The PRISMA flow diagram is depicted in Fig. 1. All studies were retrospective observational cohort studies presenting data collected between 1986 and 2015 (Table 1). Bias assessment using the NOS, with scoring per study, is presented in ESM Table 2. Overall, six studies demonstrated a low risk of bias, with the remainder showing a high risk of bias, largely due to a lack of control groups or controlled study designs. Additional details of full text articles excluded as part of the systematic search process are outlined in ESM Table 3.

**Table 1:** Baseline characteristics

Study characteristics	Total studies, *n* (%) (*N*=15)
Year of publication	
1990–1999	4 (27)
2000–2009	7 (47)
2010–2019	4 (27)
Study type	
Case–control studies	0 (0)
Prospective cohort studies	0 (0)
Retrospective cohort studies	15 (100)
Country	
Austria	3 (20)
Germany	1 (7)
Italy	3 (20)
Norway	2 (13)
Poland	1 (7)
USA	5 (33)

### Participant demographics and pre-transplant characteristics

Assessment of SPKTx and KTx recipient characteristics demonstrated that, for the former, they were generally younger at the time of transplant and there was a greater proportion of female recipients (Table 2). Although the duration of type 1 diabetes prior to solid organ transplantation in both populations was similar, a greater proportion of those receiving SPKTx were pre-emptively transplanted prior to dialysis (14% vs 8%, *p*=0.02), with a longer mean duration of dialysis in KTx recipients. There was no significant difference between the two groups with regard to lipid profile pre transplant. SPKTx recipients demonstrated higher mean HbA_1c_ and more antihypertensive medication use per individual at the time of transplant than KTx recipients. Overall, a greater proportion of SPKTx recipients were on tacrolimus and mycophenolate than KTx recipients, and cyclosporin was the most commonly used immunosuppressive following KTx. This is likely a reflection of changing clinical and centre-specific practices around pancreas allograft transplantation in the time frame of the published literature. A SoF table is provided for post-transplant outcomes in ESM Table 4.

**Table 2:** Pre-transplant recipient variables and immunosuppression post transplant

Characteristic	SPKTx (*N*=564, *k*=15)	KTx (*N*=419, *k*=15)	Total (*N*=983, *k*=15)
Pre-transplant recipient variables			
Age at transplant, years	40 ± 7	48 ± 8	43 ± 7
Female recipients, %	48	40	41
Duration of type 1 diabetes, years	26 ± 7	23 ± 7	25 ± 7
Mean follow-up time, months	46 ± 23	65 ± 34	54 ± 28
Dialysis status pre transplant, *n*^a^			
Dialysis	306	193	499
Pre-emptive transplant	51	16	67
Unreported dialysis	207	210	417
Duration of dialysis, months	29 ± 23	39 ± 28	33 ± 25
Reported as median dialysis time, months (IQR)	11 (8–16)	7 (2–11)	N/A
TG level, mmol/l	2.1 ± 0.9	2.3 ± 0.9	2.2 ± 0.9
TC level, mmol/l	5.5 ± 1.8	5.2 ± 1.1	5.4 ± 1.6
HDL level, mmol/l	1.5 ± 0.6	1.2 ± 0.4	1.4 ± 0.6
LDL level, mmol/l	2.6 ± 1.4	2.8 ± 1.4	2.6 ± 1.4
Number of antihypertensive medications per patient	2.5 ± 1.1	2.2 ± 1.3	2.4 ± 1.2
HbA_1c_, mmol/mol	74 ± 16	53 ± 10	70 ± 16
HbA_1c_, %	8.9 ± 1.9	7.0 ± 1.3	8.6 ± 1.9
Creatinine, μmol/l	156 ± 21	210 ± 29	167 ± 23
eGFR, ml/min per 1.73 m^2^	78 ± 17	69 ± 13	76 ± 16
BMI, kg/m^2^	24.7 ± 3.7	24.7 ± 4.4	24.8 ± 3.8
Immunosuppression post transplant^b^			
Azathioprine, *n* (%)	58 (10)	37 (9)	95 (10)
Cyclosporin, *n* (%)	78 (14)	112 (27)	190 (19)
Mycophenolate, *n* (%)	164 (29)	50 (12)	214 (22)
Prednisolone, *n* (%)	91 (16)	58 (14)	149 (15)
Tacrolimus, *n* (%)	180 (32)	77 (18)	257 (26)
Immunosuppression unreported, *n*	306 (*k*=8)	258 (*k*=8)	564 (*k*=8)

Continuous variables are demonstrated as mean ± SD unless otherwise specified, with categorical variables presented as frequency and percentage. Mean pre-transplant variables are presented as pooled statistics where possible

*k* represents number of studies

^a^Dialysis type (haemodialysis vs peritoneal dialysis) was not specified, except by Ziaja et al 2016 [35] (all haemodialysis patients)

^b^Immunosuppression is presented as the total number of individuals on the agents as described, and as a percentage of the total reported individuals on immunosuppression; individuals were commonly also on multiple combinations of agents

N/A, not applicable

### Lipid profile

**Fig. 2:**
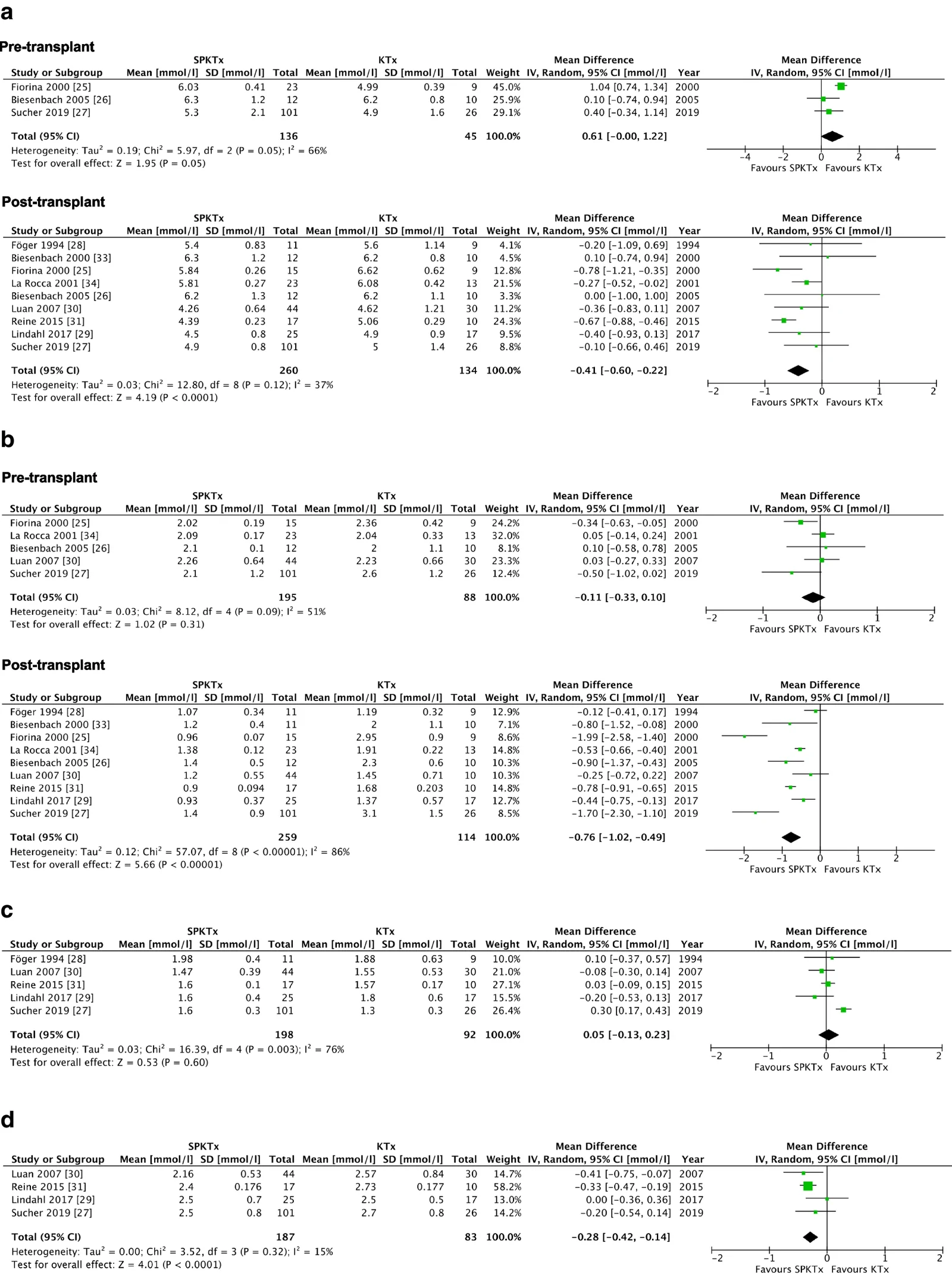
Forest plots comparing mean differences in (**a**) TC levels and (**b**) TG levels pre and post transplant between SPKTx and KTx recipients with type 1 diabetes. Post-transplant (**c**) HDL and (**d**) LDL are also depicted across the two groups. All mean and SD lipid profile measures are represented in mmol/l. IV, inverse variance

Pre-transplant TC levels were higher in SPKTx recipients than in KTx recipients, but were not statistically different (mean difference 0.61 mmol/l [95% CI 0.00, 1.22], *p*=0.05; Fig. 2a) [25–27]. Meta-analysis demonstrated significantly lower TC levels in the SPKTx group post transplant than in the KTx group (mean difference −0.41 mmol/l [95% CI −0.60, −0.22], *p*<0.01; Fig. 2a). There was no significant difference between pre- and post-transplant TC levels in KTx recipients; however, post-transplant TC was significantly lower than pre-transplant levels in SPKTx recipients (*p*<0.01).

Similar to TC levels, pre-transplant meta-analysis demonstrated no significant difference between SPKTx and KTx TG levels (mean difference −0.11 mmol/l [95% CI −0.33, 0.10], *p*=0.31; Fig. 2b). However, pooled post-transplant mean TG levels were significantly lower in the SPKTx group than in the KTx group (mean difference −0.76 mmol/l [95% CI −1.02, −0.49], *p*<0.01; Fig. 2b). As with TC levels, TG levels were not significantly different pre and post KTx; however, TG levels showed a significant decrease post SPKTx (*p*<0.01).

Pre-transplant HDL and LDL levels were poorly reported, and meta-analysis was not possible. Meta-analysis demonstrated no significant difference in post-transplant HDL levels between the SPKTx and KTx groups (mean difference 0.05 mmol/l [95% CI −0.13, 0.23], *p*=0.60; Fig. 2c) [27–31], albeit with significantly lower post-transplant LDL levels in the SPKTx group (mean difference −0.28 mmol/l [95% CI −0.42, −0.14], *p*<0.01; Fig. 2d) [27, 29–31].

### Blood pressure and HbA_1c_

**Fig. 3:**
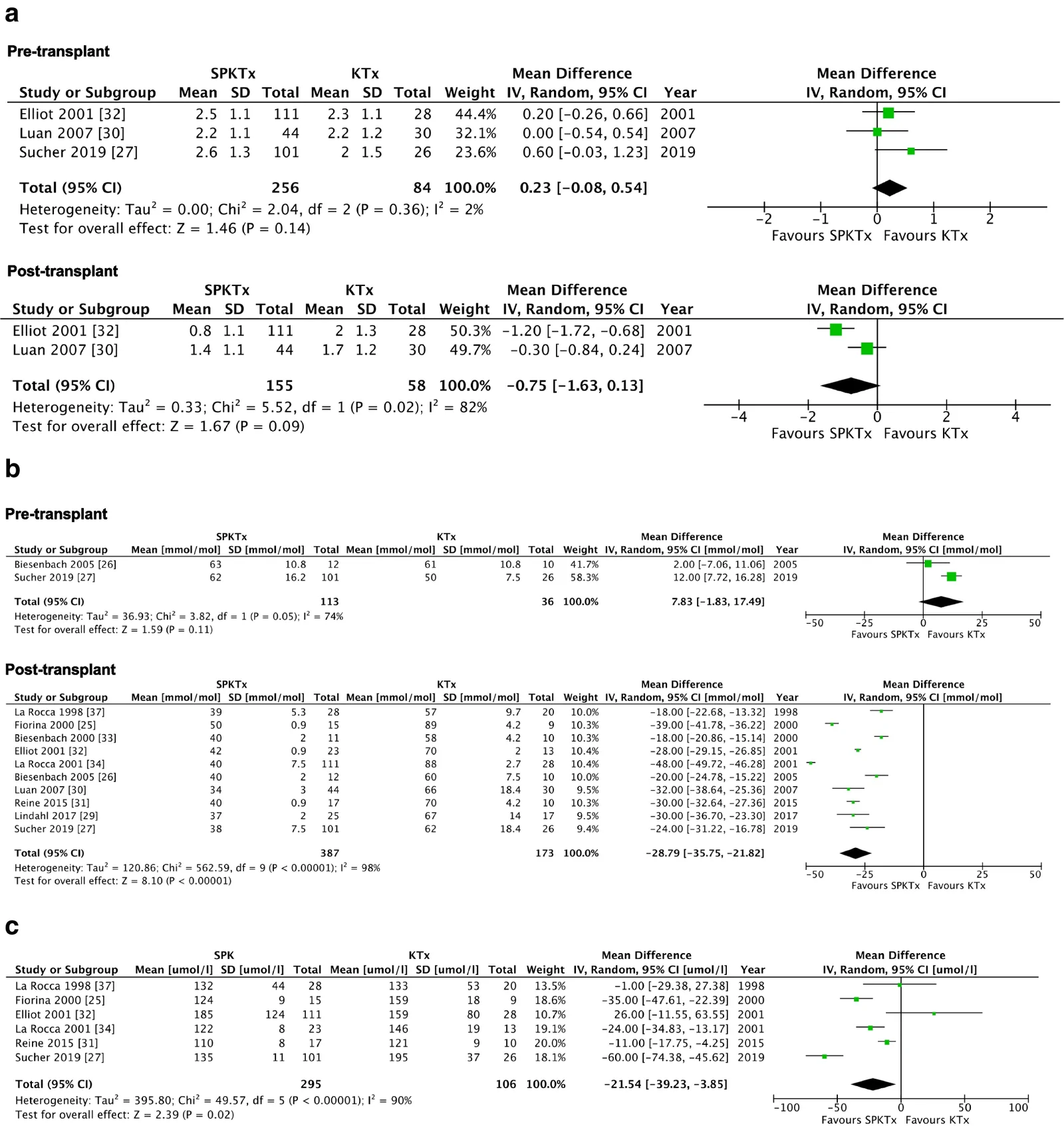
Forest plots comparing mean differences in (**a**) mean antihypertensive medication use per patient and (**b**) HbA_1c_ (mmol/mol; forest plot in % presented in ESM Fig. 1) pre and post transplant between SPKTx and KTx recipients with type 1 diabetes. (**c**) Post-transplant creatinine (μmol/l) is also depicted between the two groups. IV, inverse variance

Owing to a lack of ambulatory blood pressure reporting, our review analysed mean antihypertensive medication use per participant as a surrogate for long-term blood pressure control. There was no significant difference between mean antihypertensive use between the SPKTx and KTx groups either pre transplant (mean difference 0.23 [95% CI −0.08, 0.54], *p*=0.14; Fig. 3a) [27, 30, 32] or post transplant (mean difference −0.75 [95% CI −1.63, 0.13], *p*=0.09; Fig. 3a). However, only the SPKTx group demonstrated a significant reduction in mean antihypertensive medication use post transplantation (1.0 ± 0.6 [*n*=155]) vs pre transplant (2.5 ± 1.1 [*n*=256], *p*<0.01).

There was no significant difference in HbA_1c_ between the SPKTx and KTx groups prior to transplantation (mean difference 7.83 mmol/mol [95% CI −1.83, 17.49], *p*=0.11 [Fig. 3b]; mean difference 0.76% [95% CI −0.09, 1.62], *p*=0.11 [ESM Fig. 1]). SPKTx demonstrated significantly lower post-transplant HbA_1c_ than KTx (mean difference −28.79 mmol/mol [95% CI −35.75, −21.82], *p*<0.01 [Fig. 3b]; mean difference −2.61% [95% CI −3.05, −2.16], *p*<0.01 [ESM Fig. 1]). HbA_1c_ profile improved between pre- and post-SPKTx groups (74 ± 9 [*n*=224] vs 39 ± 5 [*n*=387] mmol/mol, respectively, *p*<0.01; 8.9 ± 1.9% vs 5.7 ± 0.7%, respectively, *p*<0.01), whereas HbA_1c_ profile was significantly worse post KTx (69 ± 16 mmol/mol [*n*=173]) than pre KTx (53 ± 13 mmol/mol [*n*=36], *p*<0.01; 8.5 ± 1.7% vs 7.0 ± 1.4%, respectively; *p*<0.01).

### Renal function

Reporting of pre-transplant creatinine and eGFR was limited in the studies included, with only post-transplant creatinine levels consistently reported. A meta-analysis of post-transplant creatinine levels demonstrated superior renal function in SPKTx than in KTx recipients (mean difference −21.54 μmol/l [95% CI −39.23, −3.85], *p*=0.02; Fig. 3c).

### Macrovascular disease and mortality

Post-transplant vascular disease progression occurred across both the SPKTx and the KTx groups in all of the relevant disease categories (ESM Table 5). Progression of CAD was largely reported by evidence of new ischaemia on coronary imaging, myocardial infarction or the need for percutaneous coronary intervention in the recipient. Although poorly reported, CeVD progression consisted of new transient ischaemic attacks or strokes in recipients, with two studies demonstrating greater events post transplant in both the SPKTx and the KTx groups [26, 33]. Overall, the greatest progression in vascular disease was seen in PVD, with both the SPKTx and the KTx groups demonstrating a twofold increase in PVD post transplant, including new amputations, lower limb ulcers and revascularisation procedures. Within studies reporting PVD complications post transplant, 18% of SPKTx recipients and 15% of KTx recipients required lower limb amputations, with 21% of SPKTx recipients and 27% of KTx recipients requiring revascularisation procedures. Amputations accounted for 35% and revascularisation accounted for 32% of total PVD-related complications after transplantation.

**Fig. 4:**
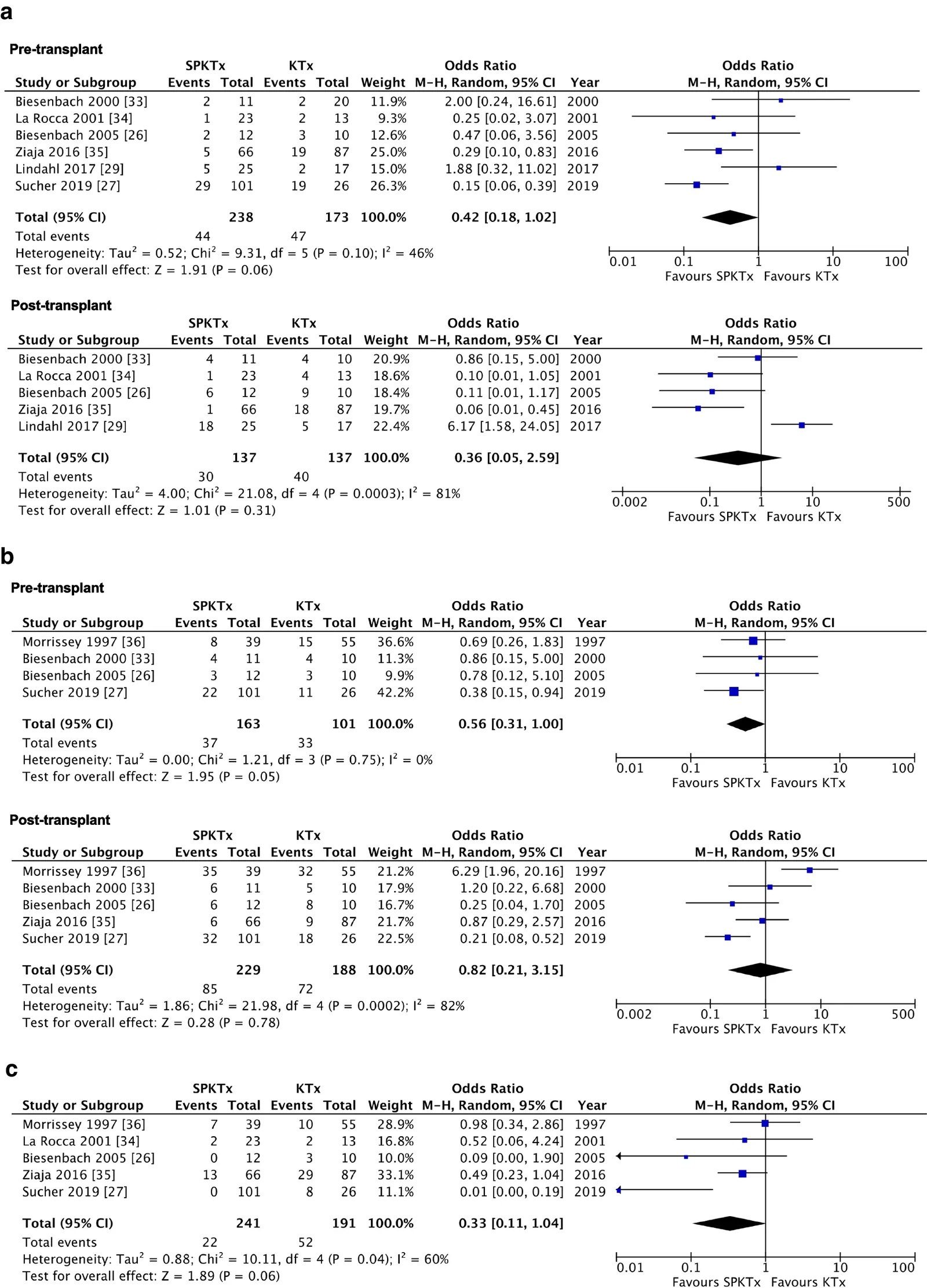
Forest plots comparing odds ratios for incidence of pre-transplant and post-transplant (**a**) CAD, (**b**) PVD and (**c**) mortality linked to macrovascular events between SPKTx and KTx recipients with type 1 diabetes. MH, Mantel-Haenszel

We conducted meta-analyses to compare the odds of pre- and post-transplant CAD and PVD in the SPKTx and KTx groups. Analysis of pre-transplant CAD demonstrated a trend towards fewer events in SPKTx recipients, although this was not significant (OR 0.42 [95% CI 0.18, 1.02], *p*=0.06; Fig. 4a) [26, 27, 29, 33–35]. There was no difference between the two groups post transplant (OR 0.36 [95% CI 0.05, 2.59], *p*=0.31; Fig. 4a). Similarly, pre-transplant PVD analysis showed a trend towards fewer events in SPKTx recipients (OR 0.56 [95% CI 0.31, 1.00], *p*=0.05; Fig. 4b), and there was no significant difference post transplant between the two groups (OR 0.82 [95% CI 0.21, 3.15], *p*=0.78; Fig. 4b) [26, 27, 33, 35, 36]. For studies reporting post-transplant mortality secondary to macrovascular causes, meta-analyses demonstrated no significant difference between the two groups, although the results suggest possible superior outcomes in the SPKTx group (OR 0.33 [95% CI 0.11, 1.04], *p*=0.06; Fig. 4c) [26, 27, 34–36].

## Discussion

This study aimed to evaluate key differences in post-transplant metabolic and vascular disease profiles between SPKTx- and KTx-based treatment of type 1 diabetes with ESKD, to guide long-term management. SPKTx conferred superior metabolic profiles with significantly lower TC, TG, LDL and HbA_1c_ post transplant than KTx. The marked improvement in lipid profile in SPKTx recipients than in KTx recipients in our study may be attributed to endogenous insulin release and direct delivery to the liver (vs exogenous administration, which acts on peripheral tissues first and is therefore less effective at regulating hepatic lipid production), although this assumes consistent surgical technique across the studies with drainage into the portal circulation vs iliac veins. Previous work has demonstrated that insulin attenuates lipolysis, while promoting triacylglycerol deposition in available adipose tissue via multiple molecular pathways [40]. We have previously published work showing that SPKTx also improves insulin sensitivity, as measured by HOMA-IR [14], which would also limit TG production, circulating free fatty acids and lipolysis. Reductions in glycaemic variability associated with SPKTx also increase hepatic TG synthesis and modify lipoprotein composition [41]. These results agree strongly with previous studies with type 1 diabetes recipients showing improvements in overall lipid profile demonstrated in SPKTx recipients up to 10 years following transplantation [14]. However, neither of the transplant modalities substantially prevented vascular disease progression. New or adverse events related to PVD post transplant accounted for the largest burden of disease across both groups. No significant difference in post-transplant mortality secondary to macrovascular events was observed between the SPKTx and KTx groups.

As expected, HbA_1c_ showed significant improvement post SPKTx, with a significantly lower mean HbA_1c_ than in KTx recipients (39 vs 69 mg/mmol). This was likely due to endogenous insulin release. In our study, KTx recipients demonstrated higher HbA_1c_ levels post transplant (69 mg/mmol) than pre transplant (53 mg/mmol). Poorer long-term glycaemic management may be explained by insulin resistance due to exposure to exogenous steroids and/or by beta cell toxicity from CNI- and mTORi-based immunosuppression agents [42]. Lifestyle factors not captured within the scope of this study may also provide important insights into glycaemic management. Diet [43], weight loss [44] and physical activity [45] are efficacious in the general population, although evidence of effectiveness following transplantation is lacking [46]. Similarly, effects on post-transplant peripheral neuropathy, retinopathy and autonomic nerve function are important microvascular outcomes related to glycaemic management [47], and these warrant further investigation in transplant recipients given their significant functional implications and effects on quality on life.

The average number of antihypertensive medications used by SPKTx recipients was lower following transplantation; there were no significant differences compared with KTx recipients. However, the lack of information on the exact antihypertensive medication and doses used is a further limitation and adds uncertainty to this analysis. In part, differences in antihypertensive use may be explained by the prolonged burden of pre-existing cardiovascular disease and/or the duration of ESKD prior to transplantation. Prior to transplantation, recipients from both the SPKTx and the KTx groups required multiple agents to manage blood pressure, presumably linked to long-standing changes from endothelial dysfunction and local renal vascular and hormonal changes associated with ESKD [48, 49]. ESKD predisposes to left ventricular hypertrophy with a direct correlation between the severity of renal dysfunction and the extent of cardiac hypertrophy [50]. Current evidence suggests that transplantation does not limit the progression of cardiac hypertrophy compared with individuals who undertake dialysis in the absence of stringent blood pressure control [51]. Additionally, CNI-based immunosuppression common to both groups may contribute to the further risk of hypertension [42].

Renal function following SPKTx was significantly better than in the KTx group, which is in keeping with previous studies demonstrating superior long-term allograft function in SPKTx recipients [52], likely reflecting younger donor age [7]. Superior long-term glycaemic management offered by the pancreatic allograft in SPKTx recipients further preserves renal allograft function and may also account for these findings [53]. Pancreas allografts also improve all-cause mortality by >30% compared with deceased kidney donor recipients [9]. The study also showed that living donor transplant outcomes may approach those of SPKTx recipients, highlighting the importance of donor organ quality in determining post-transplant outcomes. Future studies should conduct additional donor and organ selection analyses and interrogate the impact of donor status on recipient outcomes. Renal function has also been linked to overall cardiovascular outcomes post transplant [54, 55], reinforcing the concept of complex relationships between metabolic parameters and signalling the need for multi-risk-factor optimisation to improve recipient outcomes.

Progression of vascular disease was observed in both the SPKTx and the KTx groups post transplant, despite greater improvements in metabolic profile following SPKTx. Currently, there is a paucity of data that link the duration of type 1 diabetes to vascular disease outcomes in transplant recipients. Other important factors not extensively evaluated by the studies included were rates of rejection and graft dysfunction, which, by virtue of treatment and the impact on allograft longevity and the subsequent return to insulin requirement, may portend the risk of metabolic or vascular disease progression [54, 56, 57]. Progression of vascular disease may be explained by multiple factors. First, the strong links between cardiovascular risk, dialysis and ESKD may be difficult to reverse, even with solid organ transplantation [58]. Second, pre-existing vascular disease may have a significant impact on post-transplant outcomes. This is supported by findings in small, primary studies indicating that pre-existing PVD, the duration of dialysis, and kidney allograft failure remain important predictors of adverse events associated with post-transplant PVD [59]. Given these findings, the optimisation of pre-existing PVD and of the cardiovascular profile are important factors to consider in reducing overall morbidity and mortality associated with vascular disease post transplant. However, it still remains unclear whether transplantation, and specifically the normalisation of renal function, slows the progression of vascular disease. Although they were not possible to conduct in this review, subgroup comparisons including differences in outcomes related to pre-emptive transplantation, or analysis with stratification by severity of pre-existing vascular burden, would allow more robust conclusions to be made with regard to post-transplant vascular disease risk.

This systematic review was limited by a lack of data for subgroup analysis (e.g. pre-emptive vs dialysis dependent, deceased vs live donor transplantation, and pancreas allograft function vs failure), as outcomes of desired subgroups were often unreported in the studies included. Donor quality is a crucial determinant of allograft (and patient) longevity, and SPKTx recipients typically receive younger organs than KTx recipients of either living or deceased donors’ kidneys [60], although studies vary in the demonstration of survival benefits [60–62]. Heterogeneity and a lack of randomisation by virtue of the observational cohort study design of the studies included were further limitations of our meta-analysis. A minor overlap in study data may be present in some studies [26, 33, 34, 37] in which recipients were assessed over a similar time frame. However, no reference to data overlap between studies was reported, and we could not confirm the years across which the cohort data were collected in the earlier publication. Therefore, all studies were included in the analysis, as each reported different outcome measures relevant to this systematic review, preventing significant data loss through the exclusion of studies.

Important factors that we could not evaluate in parallel included medication compliance, lifestyle factors and additional outcomes such as graft function and rates of rejection. Reasons for mortality (with or without vascular disease) were not clearly outlined by the relevant studies. Furthermore, the studies identified in our systematic review were conducted across a long time period (1986 to 2015), which incorporated an evolution of surgical technique for SPKTx (enteric drainage of the pancreas allograft), but did not necessarily reflect widespread changes in immunosuppression. Due to limited data, we were not able to assess for heterogeneity in outcomes over time from practice changes pertaining to metabolic profiling and surveillance, advances in the treatment of type 1 diabetes, the evolution of immunosuppression regimens (which in turn may have impacted metabolic profile and insulin resistance) and differences in immunosuppression prescribing practices (including steroid-free protocols).

### Conclusion

This meta-analysis is the most comprehensive comparison of post-transplant metabolic and vascular outcomes in individuals with type 1 diabetes receiving SPKTx or KTx. While SPKTx is associated with improved metabolic profiles after transplantation, these benefits did not translate into clear reductions in vascular disease risk, and evidence for a mortality advantage related to macrovascular disease remains limited. Ongoing cardiovascular risk highlights the need for aggressive risk factor modification in this population. Future studies will require detailed subgroup analyses to clarify whether superior metabolic control after SPKTx meaningfully reduces vascular disease risk.

## Supplementary Information

Below is the link to the electronic supplementary material.

ESM (PDF 662 KB)

## Data Availability

The majority of data supporting the findings of this study are available within the paper and its ESM. The raw data, prior to statistical analysis, are not openly available; however, they can be made accessible from the authors on request for clearly outlined purposes from reputed sources. Data can be made available to researchers requiring further clarification of methodology where appropriate or where quoting/referencing the article where data have not been presented already.

## Abbreviations

CAD: Coronary artery disease
CeVD: Cerebrovascular disease
CNI: Calcineurin inhibitor
ESKD: End-stage kidney disease
KTx: Kidney transplantation alone
mTORi: Mammalian target of rapamycin inhibitor
NOS: Newcastle–Ottawa Scale
PRISMA: Preferred Reporting Items for Systematic Reviews and Meta-analyses
PVD: Peripheral vascular disease
SoF: Summary of findings
SPKTx: Simultaneous pancreas–kidney transplantation
TC: Total cholesterol
TG: Triglyceride
