# Supplementary material for: Metabolic and macrovascular outcomes following simultaneous pancreas–kidney transplantation and kidney transplantation alone in recipients with type 1 diabetes mellitus: a systematic review and meta-analysis
Source: Diabetologia. 2026 Aug 29;69(11):3196–209. doi: 10.1007/s00125-026-06843-x (PMC13627220; doi:10.1007/s00125-026-06843-x)
Supplement: Supplementary file 1 [file 125_2026_6843_MOESM1_ESM.pdf]

SUPPLEMENTARY ONLINE MATERIALS

- ESM Table 1 - Full systematic review search strategy per database
- ESM Table 2 - Bias appraisal – Newcastle Ottawa scale (NOS), with scoring per study
- ESM Table 3 - Summary of excluded full text studies, with reasons
- ESM Table 4 - Summary of findings table - GRADE certainty of evidence tool
- ESM Table 5 - Summary of macrovascular outcomes in included studies
- ESM Fig. 1 – Forest plot comparing HbA1c across SPK and KTx groups in %

ESM Table 1 Full systematic review search strategy as per database

<u>Search strategy as at 23/02/25</u>

MEDLINE via Ovid	
Search term	Results per line
1. SPK or simultaneous pancreas-kidney or pancreas-kidney or kidney-pancreas or "simultaneous pancreas kidney" or "simultaneous kidney pancreas"	4814
2. kidney transplantation/ or transplant*	923667
3. renal or kidney or "kidney only" or "kidney-only" or KTA or "kidney transplant alone" or "kidney-transplant alone"	1345809
4. 1 AND 2 AND 3	3099
5. metabolic or glucose or glycaem* or glycem* or lipid* or triglyceride* or cholesterol or HDL or LDL or BMI or weight or glycated haemoglobin or "HbA1c" or microvascular or macrovascular or stroke or heart attack or myocardial infarction or "adverse" or major adverse cardiac events or "MACE" or cardi* or athero* or peripheral vascular disease or “PAD” or “peripheral arter* disease” or “peripheral vascular disease” or “PVD”	7900360
6. 4 AND 5	1668
7. type I diabetes or diabetes mellitus or type 1 diabetes or insulin dependent diabetes mellitus or "IDDM"	615492
8. 6 AND 7	749
9. limit 8 to (full text and human and english language and english and (adult <18 to 64 years> or aged <65+ years>))	**414**

EMBASE via Ovid	
Search term	Results per line
1. SPK or simultaneous pancreas-kidney or pancreas-kidney or kidney-pancreas or "simultaneous pancreas kidney" or "simultaneous kidney pancreas"	9109
2. kidney transplantation/ or transplant*	1340332
3. renal or kidney or "kidney only" or "kidney-only" or KTA or "kidney transplant alone" or "kidney-transplant alone"	2236823
4. 1 AND 2 AND 3	6460
5. metabolic or glucose or glycaem* or glycem* or lipid* or triglyceride* or cholesterol or HDL or LDL or BMI or weight or glycated haemoglobin or "HbA1c" or microvascular or macrovascular or stroke or heart attack or myocardial infarction or "adverse" or major adverse cardiac events or "MACE" or cardi* or athero* or peripheral vascular disease or “PAD” or “peripheral arter* disease” or “peripheral vascular disease” or “PVD”	1155360
6. 4 AND 5	2881
7. type I diabetes or diabetes mellitus or type 1 diabetes or insulin dependent diabetes mellitus or "IDDM"	1486819
8. 6 AND 7	1532
9. limit 8 to (full text and human and english language and english and (adult <18 to 64 years> or aged <65+ years>))	**579**

SCOPUS	
Search term	Results per line
1. TITLE-ABS-KEY (spk OR simultaneous AND pancreas-kidney OR pancreas-kidney OR kidney-pancreas OR "simultaneous pancreas kidney" OR "simultaneous kidney pancreas")	7918
2. TITLE-ABS-KEY ("kidney transplantation" OR transplant*)	1177062
3. TITLE-ABS-KEY (kidney OR "kidney only" OR "kidney-only" OR kta OR "kidney transplant alone" OR "kidney-transplant alone")	1620246
4. TITLE-ABS-KEY (metabolic OR glucose OR glycaem* OR glycem* OR lipid* OR triglyceride* OR cholesterol OR "HDL" OR "LDL" OR bmi OR weight OR "glycated haemoglobin" OR "HbA1c" OR microvascular OR macrovascular OR stroke OR "heart attack" OR "myocardial infarction" OR "adverse" OR "major adverse cardiac events" OR "MACE" OR cardi* OR athero* OR "peripheral vascular disease" OR "PAD" OR "peripheral arterial disease" OR "albumin-creatinine ratio" OR acr)	10816515
5. TITLE-ABS-KEY ("type I diabetes" OR "diabetes mellitus" OR "type 1 diabetes" OR "insulin-dependent diabetes mellitus" OR "IDDM")	1125360
6. 1 AND 2 AND 3 AND 4 AND 5	1247
7. (LIMIT-TO (DOCTYPE , "ar")) AND (LIMIT-TO (LANGUAGE , "English")) AND (LIMIT-TO (EXACTKEYWORD , "Human"))	**708**

ESM Table 2 *Bias appraisal using the Newcastle-Ottawa Scale (NOS) for studies included in the systematic review.*

	SELECTION				COMPARABILITY[a]	OUTCOME			OVERALL RISK OF BIAS[b]
	Representativeness of the exposed cohort	Selection of nonexposed cohort	Ascertainment of exposure	Demonstration that outcome of interest was not present at start of study		Assessment of outcome	Follow up long enough for outcomes	Adequacy of follow up	
Biesenbach et al. 2000			*	*		*	*	*	HIGH
Biesenbach et al. 2005		*	*	*	*	*	*	*	LOW
el-Gebely et al. 1995		*	*	*		*	*	*	HIGH
Elliot et al. 2001	*		*	*		*	*	*	HIGH
Fiorina et al. 2000		*	*	*		*	*		HIGH
Foger et al. 1994	*		*	*	*	*	*	*	LOW
Knight et al. 2004	*		*	*		*	*	*	HIGH
La Rocca et al. 1998			*	*		*		*	HIGH
La Rocca et al. 2001			*	*	*	*	*	*	LOW
Lindahl et al. 2017	*		*	*		*	*	*	HIGH
Luan et al. 2007	*		*	*	*	*	*	*	LOW
Morrissey et al. 1997	*		*	*		*	*	*	HIGH
Reine et al. 2015			*	*	*	*	*	*	HIGH
Sucher et al. 2019	*		*	*	*	*	*	*	LOW
Ziaja et al. 2016	*		*	*	*	*	*	*	LOW

[a] *Two possible stars available for comparability – one star each for 1) controlling age, sex and marital status, and 2) for controlling some other factors*

[b] *Quality of studies adjudged by total stars for each study as per the following criteria - ≥7 as LOW risk of bias, 4-6 as HIGH risk, and ≤3 as VERY HIGH risk of bias – refer to Supplementary material for additional detail concerning bias assessment.*

Newcastle-Ottawa Scale quality assessment tool for observational studies – scoring per study

The Newcastle-Ottawa scale for observational studies includes 3 major categories: **1) Selection**, **2) Comparability** and **3) Outcome**, of which there are subcategories and criteria attributing stars to a maximum of 9 stars overall.
Stars are attributed to each category as follows:

1) **Selection**: maximum of 4 stars within the subcategories of:
    - Representativeness of the cohort (max 1 star):
        - If the study subjects are truly OR somewhat representative of the exposed cohort
    - Selection of non-exposed cohort (max 1 star):
        - If control groups are drawn from the same community as exposed cohorts
    - Ascertainment of exposure (max 1 star):
        - If secure record or structured interviews are used to ascertain outcomes (compared to written self-reports or no mentions/descriptions of ascertainment of exposure.)
    - Demonstration that outcome of interest was not present at the start of the study (max 1 star):
        - If yes
2) **Comparability**: maximum of 2 stars for the following:
    - Study controls for age, sex, and marital status (1 star)
    - Study controls for other factors (1 star)
3) **Outcome**: maximum of 3 stars for the following:
    - Assessment of outcome (max 1 star):
        - If Independent blind assessment OR record linkage to assess outcome
    - Was follow-up long enough for outcomes to occur (max 1 star)
        - If yes (dependent on clinical context)
    - Adequacy of follow-up of cohorts (max 1 star):
        - If complete follow-up with all subjects accounted for OR subjects lost to follow-up unlikely to introduce bias (<20% lost, or those lost on follow-up are no different to those followed).

Overall methodological quality and risk of bias of studies adjudged by the following criteria - ≥7 as LOW risk of bias, 4-6 as HIGH risk, and ≤3 as VERY HIGH risk of bias

<u>Scoring by study</u>

<u>Biesenbach et al. 2000</u>

A) Selection: maximum of 4 stars within the subcategories of:

- Representativeness of the cohort (max 1 star): *0/1; small sample size not representative of vast majority of transplant recipients*
- Selection of non-exposed cohort (max 1 star): *0/1; No control groups*
- Ascertainment of exposure (max 1 star): *1/1; Secure records were used from single hospital centre to assess outcomes, with data linkage*
- Demonstration that outcome of interest was not present at the start of the study (max 1 star): *1/1; outcomes of interest measured post intervention – either SPK or KTx*

B) Comparability: maximum of 2 stars for the following:

- Study controls for age, sex, and marital status (1 star): *0/1 – unable to control for confounders given nature of study design*
- Study controls for other factors (1 star): *0/1 – unable to control for additional factors*

C) Outcome: maximum of 3 stars for the following:

- Assessment of outcome (max 1 star): *1/1 no independent blind assessment, but record linkage utilised to assess outcome*
- Was follow-up long enough for outcomes to occur (max 1 star): *1/1 – follow up in excess of 5 years – adequate to show changes in metabolic profile*

- Adequacy of follow-up of cohorts (max 1 star): *1/1: adequate follow up, no attrition of follow up subjects/recipients*

Overall methodological quality and risk of bias: **5/9 – HIGH RISK**

Biesenbach et al. 2005

A) Selection: maximum of 4 stars within the subcategories of:

- Representativeness of the cohort (max 1 star): *0/1; small sample size not representative of vast majority of transplant recipients*
- Selection of non-exposed cohort (max 1 star): *1/1; SPK vs KTx*, *both cohorts fairly similar in selection based on pre-existing duration of diabetes*
- *Ascertainment of exposure (max 1 star): 1/1; Secure records were used from single hospital centre to assess outcomes, with data linkage*
- Demonstration that outcome of interest was not present at the start of the study (max 1 star): *1/1; outcomes of interest measured post intervention – either SPK or KTx*

B) Comparability: maximum of 2 stars for the following:

- Study controls for age, sex, and marital status (1 star): *0/1 – unable to control for all confounders given nature of study design*
- Study controls for other factors (1 star): *1/1 – controls for duration of diabetes, follow up and insulin requirements*

C) Outcome: maximum of 3 stars for the following:

- Assessment of outcome (max 1 star): *1/1 no independent blind assessment, but record linkage utilised to assess outcome*
- Was follow-up long enough for outcomes to occur (max 1 star): *1/1 – follow up in excess of 10 years, adequate to assess changes in outcomes of interest*
- Adequacy of follow-up of cohorts (max 1 star): *1/1: adequate follow up, no attrition of follow up subjects/recipients*

Overall methodological quality and risk of bias: **7/9 – LOW RISK**

El-Gebely et al. 1995

A) Selection: maximum of 4 stars within the subcategories of:

- Representativeness of the cohort (max 1 star): *0/1; small sample size not representative of vast majority of transplant recipients*
- Selection of non-exposed cohort (max 1 star): *1/1; Control group explicitly stated as KTx group who were also uremic type I diabetes candidates having received a transplant at the same time as SPK*
- Ascertainment of exposure (max 1 star): *1/1; Secure records were used from single hospital centre to assess outcomes, with data linkage*
- Demonstration that outcome of interest was not present at the start of the study (max 1 star): *1/1; outcomes of interest measured post intervention – either SPK or KTx*

B) Comparability: maximum of 2 stars for the following:

- Study controls for age, sex, and marital status (1 star): *0/1 – unable to control for confounders given nature of study design*
- Study controls for other factors (1 star): *0/1 – unable to control for additional factors*

C) Outcome: maximum of 3 stars for the following:

- Assessment of outcome (max 1 star): *1/1 no independent blind assessment, but record linkage utilised to assess outcome*
- Was follow-up long enough for outcomes to occur (max 1 star): *1/1 – follow up to 2 years post-transplant and exclusion of minimal recipients based on <2 year follow*

- Adequacy of follow-up of cohorts (max 1 star): *1/1: adequate follow up, minimal attrition of follow up subjects/recipients – <5% each group*

Overall methodological quality and risk of bias: **6/9 – HIGH RISK**

Elliot et al. 2001

A) Selection: maximum of 4 stars within the subcategories of:
- Representativeness of the cohort (max 1 star): *1/1; large sample size of SPK and KTx recipients assessed over time*
- Selection of non-exposed cohort (max 1 star): *0/1; no obvious control group outlined*
- Ascertainment of exposure (max 1 star): *1/1; Secure records were used from single hospital centre to assess outcomes, with data linkage*
- Demonstration that outcome of interest was not present at the start of the study (max 1 star): *1/1; outcomes of interest measured post intervention – either SPK or KTx*

B) Comparability: maximum of 2 stars for the following:
- Study controls for age, sex, and marital status (1 star): *0/1 – unable to control for confounders given nature of study design*
- Study controls for other factors (1 star): *0/1 – unable to control for additional factors*

C) Outcome: maximum of 3 stars for the following:
- Assessment of outcome (max 1 star): *1/1 no independent blind assessment, but record linkage utilised to assess outcome*
- Was follow-up long enough for outcomes to occur (max 1 star): *1/1 – follow up to 18 months post transplant on average*
- Adequacy of follow-up of cohorts (max 1 star): *1/1: adequate follow up, minimal attrition of follow up subjects/recipients*

Overall methodological quality and risk of bias: **6/9 – HIGH RISK**

Fiorina et al. 2000

A) Selection: maximum of 4 stars within the subcategories of:
- Representativeness of the cohort (max 1 star): *0/1; small sample size selected for review*
- Selection of non-exposed cohort (max 1 star): *1/1; clear outline that kidney transplant were considered control or non-exposed group*
- Ascertainment of exposure (max 1 star): *1/1; Secure records were used however unclear whether from a single or multiple hospitals*
- Demonstration that outcome of interest was not present at the start of the study (max 1 star): *1/1; outcomes of interest measured post intervention – either SPK or KTx*

B) Comparability: maximum of 2 stars for the following:
- Study controls for age, sex, and marital status (1 star): *0/1 – unable to control for confounders given nature of study design*
- Study controls for other factors (1 star): *0/1 – unable to control for additional factors*

C) Outcome: maximum of 3 stars for the following:
- Assessment of outcome (max 1 star): *1/1 no independent blind assessment, but record linkage utilised to assess outcome*
- Was follow-up long enough for outcomes to occur (max 1 star): *1/1 – follow up to 4 years post transplant*
- Adequacy of follow-up of cohorts (max 1 star): *0/1: some groups had substantial loss to follow up i.e. 15 recipients compared to 42 initially assessed*

Overall methodological quality and risk of bias: **5/9 – HIGH RISK**

Foger et al. 1994

A) Selection: maximum of 4 stars within the subcategories of:

- Representativeness of the cohort (max 1 star): *1/1; samples from different areas/regions rather than focussing on single centre although small sample size*
- Selection of non-exposed cohort (max 1 star): *0/1; no clear control group*
- Ascertainment of exposure (max 1 star): *1/1; Secure records from multiple hospitals*
- Demonstration that outcome of interest was not present at the start of the study (max 1 star): *1/1; outcomes of interest measured post intervention – either SPK or KTx*

B) Comparability: maximum of 2 stars for the following:

- Study controls for age, sex, and marital status (1 star): *0/1 – unable to control for confounders given nature of study design*
- Study controls for other factors (1 star): *0/1 – unable to control for additional factors*

C) Outcome: maximum of 3 stars for the following:

- Assessment of outcome (max 1 star): *1/1 no independent blind assessment, but record linkage utilised to assess outcome*
- Was follow-up long enough for outcomes to occur (max 1 star): *1/1 – follow greater than 4 years post-transplant*
- Adequacy of follow-up of cohorts (max 1 star): *1/1: minimal documented patient attrition at follow up*

Overall methodological quality and risk of bias: **7/9 – LOW RISK**

Knight et al. 2004

A) Selection: maximum of 4 stars within the subcategories of:

- Representativeness of the cohort (max 1 star): *1/1; representative sample size i.e. 88 kidney alone and 36 SPK recipients*
- Selection of non-exposed cohort (max 1 star): *0/1; no control group*
- Ascertainment of exposure (max 1 star): *1/1; Secure records from single centre specified in study*
- Demonstration that outcome of interest was not present at the start of the study (max 1 star): *1/1; outcomes of interest measured post intervention – either SPK or KTx*

B) Comparability: maximum of 2 stars for the following:

- Study controls for age, sex, and marital status (1 star): *0/1 – unable to control for confounders given nature of study design*
- Study controls for other factors (1 star): *0/1 – unable to control for additional factors*

C) Outcome: maximum of 3 stars for the following:

- Assessment of outcome (max 1 star): *1/1 no independent blind assessment, but record linkage utilised to assess outcome*
- Was follow-up long enough for outcomes to occur (max 1 star): *1/1 – median follow up time of 45 months – adequate to demonstrate appreciable changes in outcomes of interest*
- Adequacy of follow-up of cohorts (max 1 star): *1/1: minimal documented patient attrition at follow up*

Overall methodological quality and risk of bias: **6/9 – HIGH RISK**

La Rocca et al. 1998

A) Selection: maximum of 4 stars within the subcategories of:

- Representativeness of the cohort (max 1 star): *0/1; small sample size <30 for each group*

- Selection of non-exposed cohort (max 1 star): *0/1; no control group*
- Ascertainment of exposure (max 1 star): *1/1; Secure records although unclear if single vs multiple centres*
- Demonstration that outcome of interest was not present at the start of the study (max 1 star): *1/1; outcomes of interest measured post intervention – either SPK or KTx*

B) Comparability: maximum of 2 stars for the following:

- Study controls for age, sex, and marital status (1 star): *0/1 – unable to control for confounders given nature of study design*
- Study controls for other factors (1 star): *0/1 – unable to control for additional factors*

C) Outcome: maximum of 3 stars for the following:

- Assessment of outcome (max 1 star): *1/1 no independent blind assessment, but record linkage utilised to assess outcome*
- Was follow-up long enough for outcomes to occur (max 1 star): *0/1 – follow up time of 12 months for recipients*
- Adequacy of follow-up of cohorts (max 1 star): *1/1: minimal documented patient attrition at follow up*

Overall methodological quality and risk of bias: **4/9 – HIGH RISK**

La Rocca et al. 2001

A) Selection: maximum of 4 stars within the subcategories of:

- Representativeness of the cohort (max 1 star): *0/1; small sample size <30 for each group*
- Selection of non-exposed cohort (max 1 star): *0/1; no control group*
- Ascertainment of exposure (max 1 star): *1/1; Secure records although unclear if single vs multiple centres*
- Demonstration that outcome of interest was not present at the start of the study (max 1 star): *1/1; outcomes of interest measured post intervention – either SPK or KTx*

B) Comparability: maximum of 2 stars for the following:

- Study controls for age, sex, and marital status (1 star): *0/1 – unable to control for confounders given nature of study design*
- Study controls for other factors (1 star): *1/1 – attempts made to control diet, assays utilised for assessment and achieving therapeutic immunosuppression levels – all contributing to metabolic profile as potential confounders*

C) Outcome: maximum of 3 stars for the following:

- Assessment of outcome (max 1 star): *1/1 no independent blind assessment, but record linkage utilised to assess outcome*
- Was follow-up long enough for outcomes to occur (max 1 star): *1/1 – follow up across 4 years*
- Adequacy of follow-up of cohorts (max 1 star): *1/1: minimal documented patient attrition at follow up*

Overall methodological quality and risk of bias: **6/9 – HIGH RISK**

Lindahl et al. 2017

A) Selection: maximum of 4 stars within the subcategories of:

- Representativeness of the cohort (max 1 star): *1/1; small sample size, however selected from a large proportion of eligible and representative group*
- Selection of non-exposed cohort (max 1 star): *0/1; no control group*

- Ascertainment of exposure (max 1 star): *1/1; Secure records from a single centre in Norway – all transplants conducted at this centre*
- Demonstration that outcome of interest was not present at the start of the study (max 1 star): *1/1; outcomes of interest measured post intervention – either SPK or KTx*

B) Comparability: maximum of 2 stars for the following:

- Study controls for age, sex, and marital status (1 star): *0/1 – unable to control for confounders given nature of study design*
- Study controls for other factors (1 star): *0/1 – nil further confounding factors controlled for in study subject selection*

C) Outcome: maximum of 3 stars for the following:

- Assessment of outcome (max 1 star): *1/1 no independent blind assessment, but record linkage utilised to assess outcome*
- Was follow-up long enough for outcomes to occur (max 1 star): *1/1 – median follow up greater than 10 years*
- Adequacy of follow-up of cohorts (max 1 star): *1/1: no patient lost to follow up*

Overall methodological quality and risk of bias: **6/9 – HIGH RISK**

<u>Luan et al. 2017</u>

A) Selection: maximum of 4 stars within the subcategories of:

- Representativeness of the cohort (max 1 star): *1/1; small sample size, however selected from a large proportion of eligible and representative group*
- Selection of non-exposed cohort (max 1 star): *0/1; no control group*
- Ascertainment of exposure (max 1 star): *1/1; Secure records from single centre observational study*
- Demonstration that outcome of interest was not present at the start of the study (max 1 star): *1/1; outcomes of interest measured post intervention – either SPK or KTx*

B) Comparability: maximum of 2 stars for the following:

- Study controls for age, sex, and marital status (1 star): *0/1 – unable to control for confounders given nature of study design*
- Study controls for other factors (1 star): *1/1 – ensuring all included recipients had the same immunosuppression*

C) Outcome: maximum of 3 stars for the following:

- Assessment of outcome (max 1 star): *1/1 no independent blind assessment, but record linkage utilised to assess outcome*
- Was follow-up long enough for outcomes to occur (max 1 star): *1/1 – median follow up greater 45-50 months post-transplant*
- Adequacy of follow-up of cohorts (max 1 star): *1/1: no significant attrition of subjects on follow up*

Overall methodological quality and risk of bias: **7/9 – LOW RISK**

<u>Morrissey et al. 1997</u>

A) Selection: maximum of 4 stars within the subcategories of:

- Representativeness of the cohort (max 1 star): *1/1; sample size >30 from small single centre representative of cohorts*
- Selection of non-exposed cohort (max 1 star): *0/1; no control group*
- Ascertainment of exposure (max 1 star): *1/1; Secure records from single centre*
- Demonstration that outcome of interest was not present at the start of the study (max 1 star): *1/1; outcomes of interest measured post intervention – either SPK or KTx*

B) Comparability: maximum of 2 stars for the following:

- Study controls for age, sex, and marital status (1 star): *0/1 – unable to control for confounders given nature of study design*
- Study controls for other factors (1 star): *0/1 – no other factors explicitly controlled for*

C) Outcome: maximum of 3 stars for the following:

- Assessment of outcome (max 1 star): *1/1 no independent blind assessment, but record linkage utilised to assess outcome*
- Was follow-up long enough for outcomes to occur (max 1 star): *1/1 – follow up time >4 years for both SPK and KTx groups*
- Adequacy of follow-up of cohorts (max 1 star): *1/1: no documented attrition of subjects on follow up*

Overall methodological quality and risk of bias: **6/9 – HIGH RISK**

<u>Reine et al. 2015</u>

A) Selection: maximum of 4 stars within the subcategories of:

- Representativeness of the cohort (max 1 star): *0/1; smaller sample size selected from a larger group for another study, not necessarily representative of general SPK and KTx recipient population*
- Selection of non-exposed cohort (max 1 star): *0/1; no control group as expected based on observational nature of study*
- Ascertainment of exposure (max 1 star): *1/1; Secure records from single centre part of a larger subgroup*
- Demonstration that outcome of interest was not present at the start of the study (max 1 star): *1/1; outcomes of interest measured post intervention – either SPK or KTx*

B) Comparability: maximum of 2 stars for the following:

- Study controls for age, sex, and marital status (1 star): *0/1 – unable to control for confounders given nature of study design*
- Study controls for other factors (1 star): *1/1 – attempts to control for factors such as elevated CRP, and immunosuppression*

C) Outcome: maximum of 3 stars for the following:

- Assessment of outcome (max 1 star): *1/1 no independent blind assessment, but record linkage utilised to assess outcome*
- Was follow-up long enough for outcomes to occur (max 1 star): *1/1 – follow up time close to 10 years for both groups*
- Adequacy of follow-up of cohorts (max 1 star): *1/1: no documented attrition of subjects on follow up*

Overall methodological quality and risk of bias: **6/9 – HIGH RISK**

<u>Sucher et al. 2019</u>

A) Selection: maximum of 4 stars within the subcategories of:

- Representativeness of the cohort (max 1 star): *1/1; good sample sizes covering general SPK and KTx populations*
- Selection of non-exposed cohort (max 1 star): *0/1; no control group as expected based on observational nature of study*
- Ascertainment of exposure (max 1 star): *1/1; Secure records from single centre hospital conducting transplants*
- Demonstration that outcome of interest was not present at the start of the study (max 1 star): *1/1; outcomes of interest measured post intervention – either SPK or KTx*

B) Comparability: maximum of 2 stars for the following:

- Study controls for age, sex, and marital status (1 star): *0/1 – unable to control for confounders given nature of study design*

- Study controls for other factors (1 star): *1/1 – similar operative technique, consensus on excluded subjects and strictly protocolled immunosuppression regime, subgroup analysis on those with pre-existing CV factors*

C) Outcome: maximum of 3 stars for the following:

- Assessment of outcome (max 1 star): *1/1 no independent blind assessment, but record linkage utilised to assess outcome*
- Was follow-up long enough for outcomes to occur (max 1 star): *1/1 – median follow up time >100 months post-transplant – adequate time to demonstrate potential differences in outcomes*
- Adequacy of follow-up of cohorts (max 1 star): *1/1: <20% subjects lost to follow up*

Overall methodological quality and risk of bias: **7/9 – LOW RISK**

<u>Ziaja et al. 2016</u>

A) Selection: maximum of 4 stars within the subcategories of:

- Representativeness of the cohort (max 1 star): *1/1; adequate sample size with subjects from two regions*
- Selection of non-exposed cohort (max 1 star): *0/1; no control group as expected based on observational nature of study*
- Ascertainment of exposure (max 1 star): *1/1; Secure records from multiple centres in the same country*
- Demonstration that outcome of interest was not present at the start of the study (max 1 star): *1/1; outcomes of interest measured post intervention – either SPK or KTx*

B) Comparability: maximum of 2 stars for the following:

- Study controls for age, sex, and marital status (1 star): *0/1 – unable to control for confounders given nature of study design*
- Study controls for other factors (1 star): *1/1 – attempts to control subjects requiring insulin, diabetes disease onset prior to transplantation and protocolised pre-operative planning and operative technique*

C) Outcome: maximum of 3 stars for the following:

- Assessment of outcome (max 1 star): *1/1 no independent blind assessment, but record linkage utilised to assess outcome*
- Was follow-up long enough for outcomes to occur (max 1 star): *1/1 – follow up time ranging from 60-92 months as documented median time*
- Adequacy of follow-up of cohorts (max 1 star): *1/1: no significant documented loss of subjects to follow up (<20%)*

Overall methodological quality and risk of bias: **7/9 – LOW RISK**

ESM Table 3 *Summary of excluded full text studies with reasons*

Full text study reviewed	Reason (s) for exclusion
Douzdjian, V., Abecassis, M. M., Corry, R. J., & Hunsicker, L. G. (1994). Simultaneous pancreas-kidney versus kidney-alone transplants in diabetics: Increased risk of early cardiac death and acute rejection following pancreas transplants. *Clinical Transplantation, 8*(3 Pt 1), 246–251. https://doi.org/10.1111/j.1399-0012.1994.tb00335.x	Not reporting metabolic outcomes of interest
Douzdjian, V., Rice, J. C., Gugliuzza, K. K., Fish, J. C., & Carson, R. W. (1996). Renal allograft and patient outcome after transplantation: Pancreas-kidney versus kidney-alone transplants in type 1 diabetic patients versus kidney-alone transplants in nondiabetic patients. *American Journal of Kidney Diseases, 27*(1), 106–116. https://doi.org/10.1016/s0272-6386(96)90037-2	Not reporting outcomes of interest
Humar, A., Johnson, E. M., Gillingham, K. J., Sutherland, D. E., Payne, W. D., Dunn, D. L., Wrenshall, L. E., Najarian, J. S., Gruessner, R. W., & Matas, A. J. (1998). Venous thromboembolic complications after kidney and kidney-pancreas transplantation: A multivariate analysis. *Transplantation, 65*(2), 229–234. https://doi.org/10.1097/00007890-199801270-00015	Study discussed venous thromboembolism risk rather than PVD/other macrovascular complications
Humar, A., Johnson, E. M., Payne, W. D., Dunn, D. L., Wrenshall, L. E., Najarian, J. S., Gruessner, W. G., & Matas, A. J. (1998). The acutely ischemic extremity after kidney transplant: An approach to management. *Surgery, 123*(3), 344–350. https://doi.org/10.1016/S0039-6060(98)70065-6	Impossible to separate KTx and SPK data and very acute outcomes (within 48 hours)
Kuten, S. A., Graviss, E. A., Nguyen, D. T., Gaber, A. O., Sadhu, A. R., Simpson, E. P., Yi, S. G., Podder, H., Kagan, A., & Knight, R. J. (2021). Neurogenic orthostatic hypotension: A common complication of successful pancreas transplantation. *Transplant Direct, 7*(12), e795. https://doi.org/10.1097/TXD.0000000000001208	Study discussed orthostatic hypotension and other variables not relevant to primary review outcomes
Lange, U. G., Rademacher, S., Zirnstein, B., Sucher, R., Semmling, K., Bobbert, P., Lederer, A. A., Buchloh, D., Seidemann, L., Seehofer, D., Jahn, N., & Hau, H. M. (2021). Cardiovascular outcomes after simultaneous pancreas kidney transplantation compared to kidney transplantation alone: A propensity score matching analysis. *BMC Nephrology, 22*(1), 347. https://doi.org/10.1186/s12882-021-02522-8	Impossible to separate T1DM from T2DM results for HbA1c, BMI, LDL/HDL ratio BMI, CVD, CVA and macrovascular disease
Larsen, J. L., Larson, C. E., Hirst, K., Miller, S. A., Ozaki, C. F., Taylor, R. J., & Stratta, R. J. (1992). Lipid status after combined pancreas-kidney transplantation and kidney transplantation alone in type I diabetes mellitus. *Transplantation, 54*(6), 992–996. https://doi.org/10.1097/00007890-199212000-00010	Impossible to extract relevant data due to statistical manipulation
Lim, W. H., Lok, C., Kim, S. J., Knoll, G., Shah, B., Naylor, K., McArthur, E., Luo, B., Dixon, S. N., Hawley, C., Ooi, E., Viecelli, A. K., & Wong, G. (2021). Incidence of major adverse cardiovascular events and cardiac mortality in high-risk kidney-only and simultaneous pancreas-kidney transplant recipients. *Kidney International Reports, 6*(5), 1423–1428. https://doi.org/10.1016/j.ekir.2021.02.019	Impossible to separate T1DM and T2DM recipient data
Lindahl, J. P., Hartmann, A., Aakhus, S., Endresen, K., Midtvedt, K., Holdaas, H., Leivestad, T., Horneland, R., Øyen, O., Jenssen, T. (2016). Long-term cardiovascular outcomes in type 1 diabetic patients after simultaneous pancreas and kidney transplantation compared with living donor kidney transplantation. *Diabetologia, 59*(4), 844–852. https://doi.org/10.1007/s00125-015-3853-8	Impossible to extract reported CVD, cardiovascular comorbidity presented as hazard ratios
Nikkel, L. E., Iyer, S. P., Mohan, S., Zhang, A., McMahon, D. J., Tanriover, B., Cohen, D. J., Ratner, L., Hollenbeak, C. S., Rubin, M. R., Shane, E., Nickolas, T. L., & the CURE Group (The Columbia University Renal Epidemiology Group). (2013). Pancreas-kidney transplantation is associated with reduced fracture risk compared with	Impossible to extract data from hazard ratios, and reports fracture risk outcomes only

kidney-alone transplantation in men with type 1 diabetes. *Kidney International, 83*(3), 471–478. https://doi.org/10.1038/ki.2012.430	
Orsenigo, E., Socci, C., Fiorina, P., Zuber, V., Secchi, A., Di Carlo, V., & Staudacher, C. (2005). Cardiovascular benefits of simultaneous pancreas-kidney transplant versus kidney alone transplant in diabetic patients. *Transplant Proceedings, 37*(8), 3570–3571. https://doi.org/10.1016/j.transproceed.2005.09.059	Not reporting outcomes of interest
Pérez, R., Navarro, M. D., del Castillo, D., Santamaría, R., Padillo, J., Regueiro, J. C., & Aljama, P. (2001). Simultaneous pancreas-kidney transplant compared with kidney transplant in Type I diabetic patients with end-stage renal disease. *Transplant Proceedings, 33*(7-8), 3494–3495. https://doi.org/10.1016/s0041-1345(01)02412-5.	Not reporting outcomes of interest
Rangel, E. B., Melaragno, C. S., Neves, M. D., Dib, S. A., Gonzalez, A. M., Linhares, M. M., Pacheco-Silva, A., & Sá, J. R. (2010). Family history of diabetes as a new determinant of insulin sensitivity and secretion in patients who have undergone a simultaneous pancreas-kidney transplant. *Experimental and Clinical Transplantation, 8*(1), 29–37. PMID: 20199368	Not reporting outcomes of interest
Rayhill, S. C., D'Alessandro, A. M., Odorico, J. S., Knechtle, S. J., Pirsch, J. D., Heisey, D. M., Kirk, A. D., Van der Werf, W., & Sollinger, H. W. (2000). Simultaneous pancreas-kidney transplantation and living related donor renal transplantation in patients with diabetes: Is there a difference in survival? *Annals of Surgery, 231*(3), 417–423. https://doi.org/10.1097/00000658-200003000-00015	Not reporting outcomes of interest
Sharma, A., Vas, P., Cohen, S., Patel, T., Thomas, S., Fountoulakis, N., & Karalliede, J. (2019). Clinical features and burden of new onset diabetic foot ulcers post simultaneous pancreas-kidney transplantation and kidney only transplantation. *Journal of Diabetes and its Complications, 33*(9), 662–667. https://doi.org/10.1016/j.jdiacomp.2019.05.017	Impossible to separate T1DM and T2DM recipient data
Shivaswamy, V., Stevens, R. B., Zephier, R., Zephier, M., Sun, J., Groggel, G., Erickson, J., & Larsen, J. (2008). Dyslipidemia can be controlled in diabetic as well as nondiabetic recipients after kidney transplant. *Transplantation, 85*(9), 1270–1276. https://doi.org/10.1097/TP.0b013e31816de3f6	Data immediately post-transplant with short follow up (<6months)
Tang, J., Gulyani, A., Hewawasam, E., McDonald, S., Clayton, P., Webster, A. C., Kanellis, J., & Jesudason, S. (2021). Pregnancy outcomes for simultaneous pancreas-kidney transplant recipients versus kidney transplant recipients. *Clinical Transplantation, 35*(1), e14151. https://doi.org/10.1111/ctr.14151	Reporting on pregnant population of transplant recipients – outcomes of interest in desired patient population not reported

ESM Table 4 GRADE certainty of evidence tool across all metabolic and macrovascular outcomes for included studies in the systematic review.

Outcomes	No. of participants on follow-up (studies)	Certainty of the evidence (GRADE)*	Relative effect (95% CI)	Anticipated absolute effects	
				Risk with KTx†	Risk difference with KTx
Total cholesterol (TC)	394 (9)	⊕⊕◯◯ Low	-	Mean 5.34 mmol/l	MD 0.41mmol/l lower [0.60 to 0.22mmol/l lower]
Triglycerides (TG)	373 (9)	⊕⊕◯◯ Low	-	Mean 2.34 mmol/l	MD 0.76mmol/l lower [1.02 to 0.49mmol/l lower]
LDL levels	270 (4)	⊕⊕◯◯ Low	-	Mean 2.62mmol/l	MD 0.28mmol/l lower [0.42 to 0.14mmol/l lower]
HDL levels	290 (5)	⊕◯◯◯ Very low	-	Mean 1.56mmol/l	MD 0.05mmol/l higher [0.13mmol/l lower to 0.23mmol/l higher]
Anti-hypertensive medications per patient	213 (2)	⊕◯◯◯ Very low	-	Mean 1.84	MD 0.75 lower [1.63 lower to 0.13 higher]
Creatinine levels	401 (6)	⊕⊕⊕⊕ High	-	Mean 155 μmol/L	MD 21.5 μmol/L lower [39.2 μmol lower to 3.9 μmol lower]
HbA1c levels	560 (10)	⊕⊕⊕⊕ High	-	Mean 8.47%	MD 2.61% lower (3.05% to 2.16% lower]
Coronary artery disease (CAD)	274 (5)	⊕◯◯◯ Very low	OR 0.36 (0.05 to 2.59)	292 per 1,000	73 fewer per 1,000 (256 fewer to 117 more)
Cerebrovascular disease (CeVD)	175 (2)	⊕◯◯◯ Very low	OR 0.21 (0.05 to 0.88)	134 per 1,0000	57 fewer per 1,000 (183 fewer to 70 more)
Peripheral vascular disease (PVD)	417 (5)	⊕◯◯◯ Very low	OR 0.82 (0.21 to 3.15)	383 per 1,000	12 fewer per 1,000 (72 fewer to 48 more)
Mortality related to macrovascular disease	432 (5)	⊕◯◯◯ Very low	OR 0.33 (0.11 to 1.04)	273 per 1,000	182 fewer per 1,000 (328 fewer to 37 fewer)

Key: CI: confidence interval; **MD:** mean difference; **OR:** odds ratio

GRADE Working Group grades of evidence:
High certainty: we are very confident that the true effect lies close to that of the estimate of the effect.
Moderate certainty: we are moderately confident in the effect estimate: the true effect is likely to be close to the estimate of the effect, but there is a possibility that it is substantially different.
Low certainty: our confidence in the effect estimate is limited: the true effect may be substantially different from the estimate of the effect.
Very low certainty: we have very little confidence in the effect estimate: the true effect is likely to be substantially different from the estimate of effect.

†**The risk in the intervention group** (and its 95% confidence interval) is based on the assumed risk in the comparison group and the **relative effect** of the intervention (and its 95% CI).

***GRADE certainty of evidence was judged as follows**
- TC, TG, LDL judged as low certainty in evidence unclear risk of bias with low perceived risk of imprecision, inconsistency and no publication bias anticipated. A significant difference between groups was seen with no plausible confounders that would skew effect or reduce the perceived effect – statin, immunosuppression use was fairly variable across eras

- HDL, Ant-hypertensive medications, CAD, CeCD, PVD and mortality demonstrated very low certainty in evidence owing to inherent confounding, variable recording and reporting of this data in observational studies. This included high risk of bias, inconsistency and plausible confounding with no significant differences observed between SPKTx and KTx groups.
- Only HbA1c and creatinine levels showed high certainty in evidence due to standardised reporting of post-transplant HbA1c and creatinine. Although measured at different time points post-transplant the average duration of follow up was significant enough to demonstrate adequate effect/change in these parameters post-transplant. For these measures standardised measures, and low risk of imprecision, inconsistency, indirectness and publication bias demonstrated high certainty in evidence for creatinine and HbA1c levels.

ESM Table 5 New diagnosis, complications of macrovascular disease or associated events including coronary artery disease (CAD), cerebrovascular disease (CVD) and peripheral vascular disease (PVD) both pre- and post- transplant for simultaneous pancreas-kidney (SPK) and kidney transplantation alone (KTx) represented alongside mortality related to macrovascular disease. All data is reported as number and frequency (%) of incidents requiring management or intervention, or presence of disease based on radiographic and other objective measures. Additional details pertaining to each group of macrovascular disease is provided where available. N/A – no reporting of data. MI – myocardial infarction. PCI – percutaneous coronary intervention.

**Study**	**Transplantation type (n=)**	**Mortality post-transplant, n (%)**	**Progression of macrovascular disease? (Yes/No)**	**Recipients with macrovascular disease or associated events, n (%)**					
				**CAD**		**CVD**		**PVD**	
				Pre-transplant	***Post-transplant***	***Pre-transplant***	***Post-transplant***	***Pre-transplant***	***Post-transplant***
**Morrisey et al. 1997**	SPK (n=39)	7 (18)	Yes	9 (23)	N/A	N/A	N/A	8 (21)<br>- 4 amputations,<br>- 3 lower limb ulcers,<br>- 1 revascularisations*	35 (90)<br>- 9 amputations,<br>- 11 lower limb ulcers,<br>- 15 revascularisation with 7 failed
	KTx (n=55)	10 (18)	Yes	26 (48)	N/A	N/A	N/A	15 (27)<br>- 9 amputations,<br>- 3 lower limb ulcers, - 1 revascularisations	32 (58)<br>- 10 amputations,<br>- 8 lower limb ulcers, - 14 revascularisations with 3 failed
**Biesenbach et al. 2000**	SPK (n=11)	0 (0)	Yes	2 (18)<br>- ischemic changes on ECG or angina	4 (36)<br>- 3 ischemic changes on ECG or angina<br>- 1 MI	1 (9)<br>- carotid artery stenosis	4 (36)<br>- 2 carotid artery stenosis<br>- 2 stroke	4 (36)<br>- 1 media-sclerosis or ankle/brachial pressure index 1.0-0.7<br>- 3 ankle/brachial pressure less than 0.7 with pain	6 (55)<br>- 3 media-sclerosis or ankle/brachial pressure index 1.0-0.7<br>- 1 ankle/brachial pressure index less than 0.7 without symptoms<br>- 2 ischaemic ulceration requiring amputation.
	KTx (n=10)	0 (0)	Yes	2 (20)<br>- ischemic changes on	4 (40)<br>- 1 ischemic changes on	2 (20)<br>- carotid	5 (50)<br>- 3 carotid	4 (40)<br>- 3 media-sclerosis or	5 (50)<br>- 2 ankle/brachial pressure index

				ECG or angina	ECG or stable angina<br>- ischaemic changes in ECG or unstable angina<br>- 2 MI	artery stenosis	artery stenosis<br>- 2 stroke	ankle/brachial pressure index 1.0-0.7<br>- 1 ankle/brachial pressure less than 0.7 with pain	less than 0.7 without symptoms<br>- 1 ankle/brachial pressure less than 0.7 with pain<br>- 2 ischaemic ulceration requiring amputation.
**La Rocca et al. 2001**	SPK (n=23)	2 (9)	Yes	1 (23)<br>- ischemic changes on nuclear medicine scan	1 (23)<br>- heart failure related to ischemic heart disease	N/A	N/A	N/A	N/A
	KTx (n=13)	2 (15)	Yes	2 (15)<br>- ischemic changes on nuclear medicine scan	4 (30)<br>- 1 heart failure related to ischemic heart disease<br>- 1 MI	N/A	N/A	N/A	N/A
**Biesenbach et al. 2005**	SPK (n=12)	0 (0)	Yes	2 (16)	6 (50)	3 (25)	5 (41)	3 (25)	6 (50)
	KTx (n=10)	3 (30)<br>- 2 MI<br>- 1 sepsis	Yes	3 (30)	9 (90)	3 (30)	8 (80)	3 (30)	8 (80)
**Ziaja et al. 2016**	SPK (n=66)	13 (20)	No	5 (8) previous cardiovascular disease	1 (2)<br>- cardiovascular episode	N/A	1 (2)<br>- stroke or ITA	N/A	6 (9)<br>- limb or toe amputation
	KTx (n=87)	29 (33)	No	19 (22) previous cardiovascular disease	18 (21)<br>- 11 cardiovascular episode<br>- 7 MI	N/A	5 (6)<br>- stroke or TIA	N/A	9 (10)<br>- limb or toe amputation
**Lindahl et al. 2017**	SPK (n=25)	0 (0)	Yes	5 (20)<br>- 2 PCI<br>- 3 MI	18 (72)<br>- 1 PCI<br>- 2 MI<br>- 5 coronary bypass grafts	N/A	N/A	N/A	N/A

					- 10 progressive coronary angiogram changes				
	KTx (n=17)	0 (0)	Yes	2 (12)<br>- 1 PCI<br>- 1 MI	5 (29)<br>- 5 progressive coronary angiogram changes	N/A	N/A	N/A	N/A
**Sucher et al. 2019**	SPK (n=101)	0 (0)	Yes	29 (29)	N/A	N/A	N/A	22 (22)<br>- 8 amputations,<br>- 5 lower limb ulcers<br>- 9 revascularisations	32 (32)<br>- 12 amputations,<br>- 6 lower limb ulcers,<br>- 14 revascularisations
	KTx (n=26)	8 (31)	Yes	19 (73)	N/A	N/A	N/A	11 (42)<br>- 5 amputations,<br>- 2 lower limb ulcers, - 4 revascularisations	18 (69)<br>- 6 amputations,<br>- 4 lower limb ulcers, - 8 revascularisations

*Revascularisation therapy across studies involved lower extremity angiography ± bypass for chronic limb ischemia or vascular occlusion

Pre-transplant

Study or Subgroup	SPKTx Mean [%]	SPKTx SD [%]	SPKTx Total	KTx Mean [%]	KTx SD [%]	KTx Total	Weight	Mean Difference IV, Random, 95% CI [%]	Year
Biesenbach 2005 [26]	7.9	1.2	12	7.7	1.2	10	37.4%	0.20 [−0.81, 1.21]	2005
Sucher 2019 [27]	7.8	1.7	101	6.7	0.9	26	62.6%	1.10 [0.62, 1.58]	2019
**Total (95% CI)**			**113**			**36**	**100.0%**	**0.76 [−0.09, 1.62]**	

Heterogeneity: $Tau^2 = 0.24$; $Chi^2 = 2.50$, df = 1 (P = 0.11); $I^2 = 60\%$
Test for overall effect: Z = 1.75 (P = 0.08)

Mean Difference IV, Random, 95% CI [%]

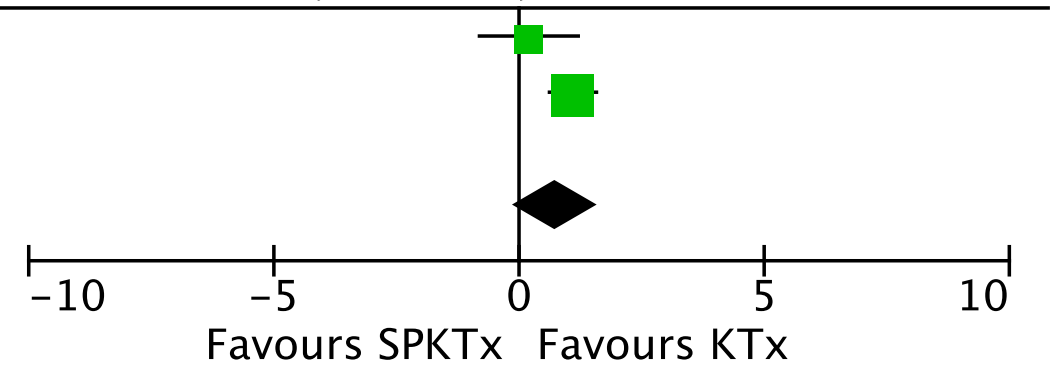

Post-transplant

Study or Subgroup	SPKTx Mean [%]	SPKTx SD [%]	SPKTx Total	KTx Mean [%]	KTx SD [%]	KTx Total	Weight	Mean Difference IV, Random, 95% CI [%]	Year
La Rocca 1998 [37]	5.7	0.7	28	7.4	1.1	20	10.2%	−1.70 [−2.25, −1.15]	1998
Biesenbach 2000 [33]	5.8	0.2	11	7.5	0.6	10	11.1%	−1.70 [−2.09, −1.31]	2000
Fiorina 2000 [25]	6.7	0.1	15	10.3	0.6	9	11.1%	−3.60 [−4.00, −3.20]	2000
La Rocca 2001 [34]	6	0.1	23	8.6	0.4	13	11.8%	−2.60 [−2.82, −2.38]	2001
Elliot 2001 [32]	5.8	0.9	111	10.2	2.7	28	7.4%	−4.40 [−5.41, −3.39]	2001
Biesenbach 2005 [26]	5.8	0.4	12	7.6	0.9	10	9.9%	−1.80 [−2.40, −1.20]	2005
Luan 2007 [30]	5.3	0.5	44	8.2	1.9	30	9.3%	−2.90 [−3.60, −2.20]	2007
Reine 2015 [31]	5.8	0.1	17	8.6	0.6	10	11.2%	−2.80 [−3.17, −2.43]	2015
Lindahl 2017 [29]	5.5	0.4	25	8.3	1.5	17	9.1%	−2.80 [−3.53, −2.07]	2017
Sucher 2019 [27]	5.6	0.9	101	7.8	1.9	26	9.0%	−2.20 [−2.95, −1.45]	2019
**Total (95% CI)**			**387**			**173**	**100.0%**	**−2.61 [−3.05, −2.16]**	

Heterogeneity: $Tau^2 = 0.42$; $Chi^2 = 77.37$, df = 9 (P < 0.00001); $I^2 = 88\%$
Test for overall effect: Z = 11.54 (P < 0.00001)

Mean Difference IV, Random, 95% CI [%]

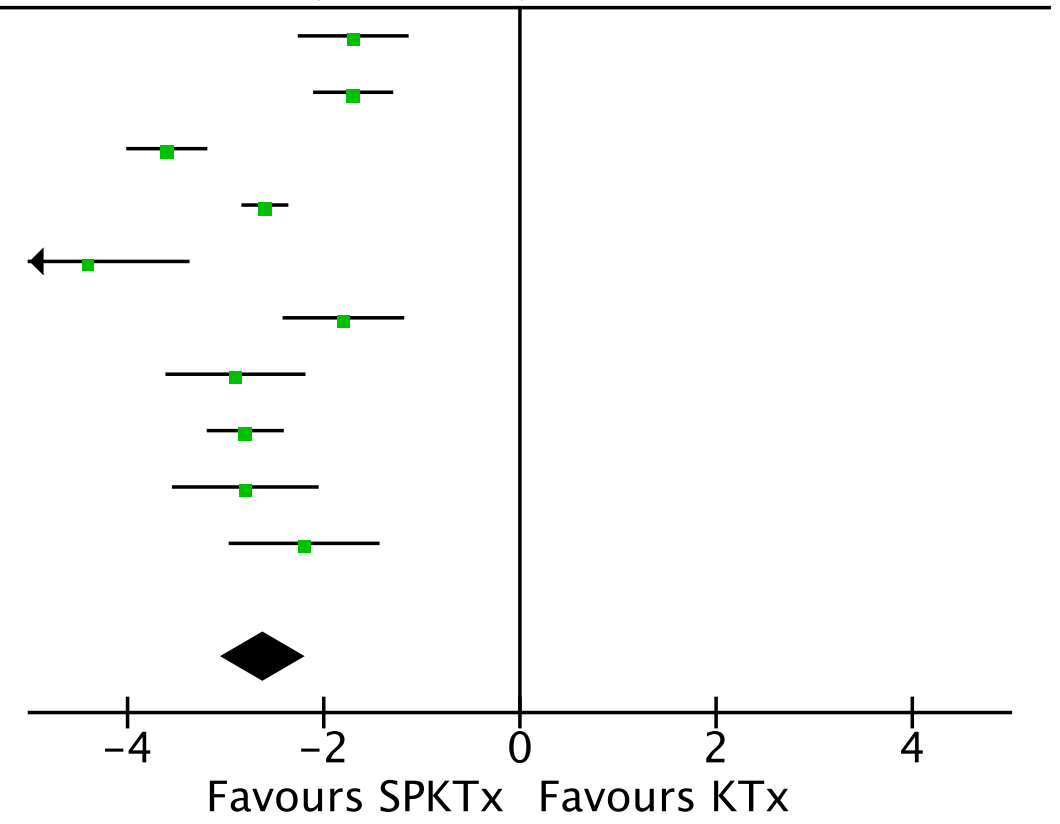

ESM Fig 1. *Forest plots comparing mean differences in HbA1c (%) pre-transplant and post-transplant between SPKTx and KTx type I diabetes recipients. SD - standard deviation; SPKTx – simultaneous pancreas-kidney transplant; KTx – kidney transplant alone; CI = confidence interval.*
